# Phthalimide conjugation turns the AIE-active tetraphenylethylene unit non-emissive: its use in turn-on sensing of hydrazine in solution and the solid- and vapour-phase†

**DOI:** 10.1039/d1ra03563k

**Published:** 2021-06-15

**Authors:** Sharanabasava D. Hiremath, Ram U. Gawas, Dharmendra Das, Viraj G. Naik, Akhil A. Bhosle, Vishnu Priya Murali, Kaustabh Kumar Maiti, Raghunath Acharya, Mainak Banerjee, Amrita Chatterjee

**Affiliations:** Department of Chemistry, BITS, Pilani – K. K. Birla Goa Campus NH 17B Bypass Road Zuarinagar Goa 403726 India amrita@goa.bits-pilani.ac.in mainak@goa.bits-pilani.ac.in; CSIR-National Institute for Interdisciplinary Science and Technology (CSIR-NIIST) Thiruvananthapuram Kerala 695019 India; Radiochemistry Division, Bhabha Atomic Research Centre Trombay Mumbai 400085 India; Department of Atomic Energy, Homi Bhabha National Institute Mumbai 400094 India

## Abstract

Hydrazine is a vital precursor used in several pharmaceuticals and pesticide industries and upon exposure can cause severe health hazards. Herein, a new AIEgen, tetraphenylethylene phthalimide (TPE-PMI), is synthesized in a one-step solvent-free mechanochemical approach exploiting the simple condensation between TPE-NH_2_ and phthalic anhydride and used for the selective and sensitive detection of hydrazine. TPE-PMI with an AIE-active TPE-moiety is non-emissive in the solid phase by design. Hydrazine performs the cleavage of TPE-PMI in a typical “Gabriel synthesis” pathway to release AIE-active TPE-NH_2_ in an aqueous solution to emit blue fluorescence. A gradual rise in fluorescence intensity at 462 nm was due to the increasing hydrazine concentration and TPE-PMI showed a linear relationship with hydrazine in the concentration range from 0.2 to 3 μM. The selectivity study confirmed that the probe is inert to amines, amino acids, metal anions, anions and even common oxidants and reductants. The detection limit is 6.4 ppb which is lower than the US Environmental Protection Agency standard (10 ppb). The practical utilities of TPE-PMI were successfully demonstrated through quantitative detection of hydrazine vapour on solid platforms like paper strips and TLC plates. Furthermore, on-site detection of hydrazine in the solid phase was demonstrated by spiking the soil samples with measured quantities of hydrazine and quantitation through image analysis. This cost-effective sensing tool was successfully utilized in *in vitro* detection of hydrazine in live HeLa cells.

## Abstract 2

A new AIE-based fluorimetric probe (TPE-PMI) has been successfully developed utilizing Gabriel reaction for the selective detection of hydrazine in solid, liquid and vapour phases.

## Graphical abstract

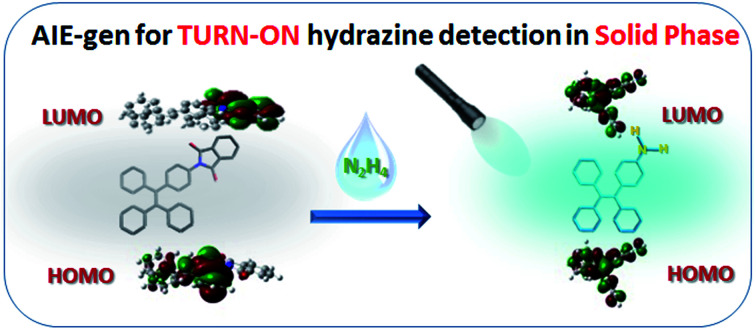

## Introduction

Hydrazine is an important chemical in different industrial sectors and is extensively used to prepare textile dyes, pharmaceuticals, polymers, pesticides, emulsifiers, polymers, *etc.*^1,2^ It is also commonly used as a rocket fuel because of its very high heat of combustion.^3^ Despite its prevalent industrial uses, hydrazine is well recognized for its severe noxiousness.^4–6^ Because of its mutagenic effect, it can harm the central nervous system and can easily get engrossed by humans affecting severe damage to liver, kidney, lungs and other organs.^7,8^ The immense usage of hydrazine leads to its contamination in soil and ground-water during the course of manufacturing, transportation, and disposal, and hence can easily enter the food chain. Owing to its high toxicity and carcinogenic nature, the US Environmental Protection Agency (US EPA) has categorized it as a group B2 chemical with a threshold limit of 10 ppb.^9^ To protect human health and the environment from the adverse effect of hydrazine, the development of sensitive, selective and cost-effective methods for its detection is necessary. Consequently, a large variety of sensing strategies have been adopted for hydrazine, including electrochemistry,^10^ surface-enhanced Raman spectroscopy,^11^ nanoparticle-based sensors,^12^ amperometry,^13^*etc.*^14^ The key limits of these strategies are the use of expensive and intricate instrumentation, lengthy data acquirement time, engrossment of skilled personnel, which causes difficulty for real-time and on-site detection and monitoring.

Fluorimetric sensing, which involves interacting analyte with compatible organic molecules, offers crucial advantages such as high sensitivity, selectivity and cost-effectiveness, and has attracted many researchers in recent times.^15,16^ A good number of chemosensors have been developed for fluorimetric detection of hydrazine^17–19^ based on well-known conventional dye molecules such as rhodols,^20^ fluorescein,^21^ BODIPY,^22^ dansyl,^23^ resorufin,^24^ coumarin,^25–27^ and many others.^28–33^ In most of these reported methods, hydrazine has been assayed by evaluating the intensity changes in the fluorescence output of the probe molecule by varying the concentration of the analyte in solution phase by the use of a fluorimeter.^17–19^ However, for the onsite analyte monitoring, it is always preferred to develop an assay system on the solid supports as it does not require expensive and sophisticated instrumentation. It is noteworthy to mention that a solid-supported assay kit with the conventional fluorescent probes is difficult to achieve as they frequently suffer from the aggregation-caused quenching (ACQ) effect, when dispersed in a suitable solvent or assimilated into solid matrices, causing a huge diminution in the performance and sensitivity.^34^ The ACQ effect invokes enormous concerns in real-life uses, predominantly, in the solid phase detection of analytes.^35^

In the recent past, aggregation-induced emission (AIE) has been exhibited by a new class of fluorogenic molecules.^35,36^ These molecular probes, called AIEgens, can overcome ACQ through emission owing to the restriction of intermolecular bond rotations (RIR) and the proscription of energy dissipation upon aggregation.^37^ They exhibit high quantum efficiency, good biocompatibility and photo-stability and therefore, find an assortment of applications in diverse fields such as chemo-/bio-sensors,^38,39^ electroluminescent materials,^40^ cell imaging,^41^ optical devices^42^*etc.*^43^ Among the reported AIE-gens, tetraphenylethylene (TPE) is one of the most studied luminophores due to its easy synthesis and simple functionalization strategies for the construction of novel sensing systems and various other applications.^38,43^ Though these AIE-gens can solve ACQ problems, the development of a turn-on type of AIE-based sensor on the solid supports remains challenging because of their auto-illuminating nature in the solid phase.^35,36^ However, from our unprecedented observation of the formation of a non-emissive TPE-azide dye in the solid phase during nitrite sensing by a TPE-NH_2_ based AIE-probe,^44^ we realized that a non-emissive TPE-probe is possible which remains turn-off even in the solid phase, by the withdrawal of electron density from an emissive TPE-NH_2_. We envisaged a phthalimide conjugated TPE (TPE-PMI) will be non-emissive in the solution phase as well as in the solid-state. Relating the information that only hydrazine could spontaneously regenerate the amino group from its phthalimide derivative by a unique “Gabriel synthesis” type cleavage to release the AIE-active unit, it paved us the way to design a highly selective sensor for hydrazine.

In the last few years, mechanochemical synthesis of organic compounds has become one of the emerging methods because it is a high yielding, energy-efficient, economic, and environmental friendly protocol that can avoid harsh conditions, use of volatile organic solvents *etc.*^45,46^ In recent years, our research efforts are directed towards designing and synthesizing fluorimetric sensors^47–51^ for environmentally and biologically important analytes particularly by environmentally benign ways^49–51^ including mechanochemistry.^51^ Herein, we report a non-emissive new AIEgen, tetraphenylethylene phthalimide (TPE-PMI), by the simple condensation of TPE-NH_2_ with phthalic anhydride using solvent-free mechanochemical condition and used for the selective and sensitive detection of hydrazine using AIE-phenomenon.

## Experimental

### Chemicals and materials

4-Aminobenzophenone, benzophenone and hydrazine monohydrate (N_2_H_4_, 64–65%) were supplied by Sigma Aldrich (India). TiCl_4_ was procured from Spectrochem Pvt. Ltd. Mumbai (India). Phthalic anhydride was purchased from SDFCL (India). All other common chemicals and solvents were obtained from local commercial suppliers and were used without further purification. All aqueous solutions were prepared with ultrapure water obtained from Millipore system and purged with N_2_ for 15 min before use.

### Instruments

NMR spectra (^1^H and ^13^C) were recorded on Bruker Avance (400 MHz) with tetramethylsilane (TMS) as the internal standard. HRMS spectra were recorded on QTOF LC-MS (6545 Q-TOF LCMS, Agilent) using ESI as an ion source. Mass spectra were obtained from Agilent 6400B LC-MS (ESI). An IR affinity-1 Fourier transform infrared (FT-IR) spectrophotometer (Shimadzu) was used to record IR spectra and KBr was used to prepare pellets. Fluorescence spectra were measured on a JASCO FP-6300 spectrofluorometer; the slit width was 2.5 nm for excitation and emission; the excitation wavelength was set to 345 nm and emission spectra were recorded in the range of 350 to 600 nm. Theoretical studies were performed with Gaussian G09W using the density functional theory (DFT) method. The fluorescence images of HeLa cells were captured using a DAPI filter of Nikon fluorescent microscope. ImageJ public domain image processing software was used for image analysis.

### Procedure for sensing studies

1 mM stock solutions of TPE-PMI and hydrazine were prepared in CH_3_CN and deionized water (MilliQ, 18 MΩ cm), respectively. The fluorescence studies were carried out by the addition of TPE-PMI (10 μM) in 3% CH_3_CN in HEPES buffer (10 mM, 7.0 pH) in a 1 mL cuvette. For selectivity study, stock solutions of chlorides and nitrates of cations (1 mM) and sodium salts of anions (1 mM), different amines and amino acids (1 mM) were prepared in deionized water. For some other organic molecules, if it is not freely soluble in water, the stock solutions were prepared in CH_3_CN (1 mM) and subsequently used for selectivity studies. The solutions for real sample analytes were prepared by spiking hydrazine in water samples collected from various water bodies (field, tap, pond, rain and river water). All solutions were subjected to filtration through 0.22 μm syringe filter to avoid any interference by any particulate matter in fluorescence measurement. All the experiments were performed three independent times at room temperature and the average data were reported.

### Synthesis of 4-(1,2,2-triphenylvinyl)benzenamine (TPE-NH_2_)

TPE-NH_2_ was prepared from an equimolar mixture of 4-aminobenzophenone and benzophenone using McMurry cross-coupling reaction according to the reported procedure^44^ and characterized by NMR (^1^H & ^13^C) and ESI-MS (see ESI for details†).

### Synthesis of 2-(4-(1,2,2-triphenylvinyl)phenyl)isoindoline-1,3-dione (TPE-PMI)

In a 5 mL stainless steel (SS) jar, TPE-NH_2_ (100 mg, 0.29 mmol) and phthalic anhydride (43 mg, 0.29 mmol) were taken with one 10 mm SS ball. The two components were milled in a Retsch MM400 mixer-mill for 1 h at 30 Hz. Next, the solid mixture was transferred to a 10 mL Teflon container and heated in a sand bath at 230 °C for 2 h. The TLC revealed complete consumption of TPE-NH_2_ and formation of a single product. The sufficiently pure TPE-PMI was recrystallized from EtOH–water to afford the probe as off-white solid (130 mg, 94%), mp 283 °C; ^1^H NMR (400 MHz, CDCl_3_), *δ* (ppm) 7.94 (2H, dd, *J*_1_ = 3.2 Hz, *J*_2_ = 5.6 Hz), 7.79 (2H, dd, *J*_1_ = 3.2 Hz, *J*_2_ = 5.6 Hz), 7.27–7.24 (2H, m), 7.22–7.17 (2H, m), 7.16–7.05 (15H, m); ^13^C NMR (100 MHz, CDCl_3_), *δ* (ppm) 167.17, 143.53, 143.34, 143.30, 143.24, 141.73, 139.95, 134.34, 131.84, 131.67, 131.41, 131.37, 131.30, 131.27, 129.78, 127.81, 127.72, 127.62, 126.69, 126.54, 126.49, 125.36, 123.63; IR (KBr): *ν* 3028, 1709, 1598, 1510, 1389 cm^−1^; HRMS (ESI): calculated for C_34_H_24_NO_2_ [M + H]^+^: 478.1729, found 478.1754.

### DFT studies

Quantum mechanical studies were performed at the density functional theory (DFT)^52^ and time-dependent density functional theory (TD-DFT)^53^ levels using the Gaussian 09 program to understand the ground and excited state behaviours of TPE-PMI and TPE-NH_2_. Becke's three-parameter exchange function (B3)^54^ with Lee–Yang–Parr correlation function (LYP)^55^ have been employed using the 6-311G basis set for the geometry optimization calculations.

### General procedure for image analysis and solid, solution, vapour phase detection of hydrazine

The TLC plates were dipped in TPE-PMI (1 mM) solution. On the dried TLC plates different concentrations of hydrazine (0, 20, 40, 80, 120 and 160 μM) were drop casted and directly subjected to image analysis by ImageJ software.^56^ For solution phase detection of hydrazine TPE-PMI (1 mM) was drop casted on culture plates and sprayed with hydrazine (1 mM) solution. In vapour phase detection, the TLC plate was engraved as “BITS” with TPE-PMI (1 mM) solution soaked earbud and was exposed to hydrazine vapour in a closed container for 30 min. For solid-phase detection, the soil samples were collected from the different landmass, spiked with hydrazine followed by the addition of TPE-PMI. In another set of study, the soil samples contaminated with hydrazine were transferred into the TPE-PMI solution. The fluorescence images were taken under long UV light source (365 nm). All the images were taken by Samsung M31 smartphone with 64 megapixels camera without any filter.

### Cytotoxicity and cell imaging studies

For cytotoxicity studies A549, HEPG2, HeLa cells and for imaging only HeLa cells were grown in Dulbecco's Modified Eagle's Medium (DMEM) supplemented with 10% (v/v) fetal bovine serum, separately. Cells were seeded (1 × 10^4^) in a 96 well plate and after 24 h, treated with various concentrations of TPE-PMI (0–200 μM) for 24 h and performed the MTT assay. For time-dependent fluorescence cell imaging, HeLa cells in log phase were harvested by trypsinization and centrifugation and seeded in a 96 well plate (8 × 10^3^ cells per well). Firstly, HeLa cells were incubated for 10–30 min in the presence of hydrazine and washed with PBS buffer. Next, the cells were treated with TPE-PMI (10 μM) for 1 h, followed by thrice PBS buffer wash. The detection of hydrazine on HeLa cells was monitored at different time-intervals (10 min, 15 min, 30 min). Next, for concentration-dependent fluorescence cell imaging, the washed HeLa cells were loaded with different concentrations of hydrazine (0, 20, 50 and 100 μM) respectively at 37 °C for 1 h. After removal of free hydrazine by washing the cells with PBS buffer, the cells were loaded with TPE-PMI (10 μM); incubated for another 1 h. For the cell imaging studies, the fluorescence and bright-field images were taken directly under a DAPI filter of Nikon fluorescent microscope, combining the phase-contrast system and the fluorescence system, without further washing steps.

## Results and discussion

### Design and synthesis of TPE-PMI

In 2015, an AIE based turn-off type probe was reported by our group for the detection of nitrite and nitrate ions utilizing well-known diazotization reaction of TPE-NH_2_.^44^ The π-extended TPE-based product, an azo dye, showed quenching property, presumably because of strong electron delocalization of the electron-rich donor (TPE unit) to the quencher group (–N=N–Ar). Several research groups have exploited this phenomenon to develop TPE-based probes for selective detection of hydrazine.^57–59^ For example, Liu and co-workers, in 2017, developed a TPE-based fluorogenic probe for naked-eye sensing of hydrazine employing a similar strategy of the attachment of an azo group (–N=N–) as the quencher unit to a suitable TPE moiety.^57^ The azo group was reduced to –NH–NH– in the presence of hydrazine to open the passage of electron flow to give a turn-on fluorescence response. However, a simple reduction of azo group can also be achieved by other reducing agents which may lead to the poor selectivity of the probe.^60^ The same group previously developed another TPE-probe with cyanovinyl group as the acceptor which showed moderate interference by glutathione.^58^ However, fluorescent probe with a cyanovinyl group for selective detection of hydrazine known to show response in the presence of nucleophiles like HSO_3_^−^,^25^ amines,^59,61^*etc.*^62^ Considering these facts, we designed a solid-state AIE-suppressed probe (TPE-PMI) for hydrazine consisting of two features: (1) tetraphenylethylene (TPE) as an AIEgen to show high fluorescence in the aggregated state and (2) phthalimide moiety as a quencher and the reactive site for hydrazine *via* “Gabriel synthesis pathway”. It was considered that by cleaving the phthalimide group, TPE-PMI switches to the fluorescence ON state by releasing TPE-NH_2_; potentially capable of detecting hydrazine in solution, vapour and solid phase with utmost selectivity. Practically, the probe was synthesized in a single step by 100% atom-economic way from a known TPE-derivative (TPE-NH_2_). First, TPE-NH_2_ was synthesized from benzophenone and 4-aminobenzophenone by McMurry cross-coupling and characterized (see ESI for details†).^44^ The TPE-NH_2_ was converted to TPE-PMI using a mechanochemical method by milling of TPE-NH_2_ and phthalic anhydride under neat condition followed by heating at 230 °C in a Teflon container resulting in the formation of TPE-PMI in high yield. The product was characterized by ^1^H and ^13^C NMR, HRMS and IR spectroscopy. The peak at *δ* (ppm) 167.17 of ^13^C and 1703 cm^−1^ of IR confirms the imide carbonyl of TPE-PMI.

### Solvent screening and spectral investigation of TPE-PMI towards hydrazine

As anticipated, TPE-PMI was non-emissive in organic solvents like CH_3_CN, DMF, DMSO and THF and did not manifest any AIE response upon varying the water fractions in the aqueous-organic solvent mixture (Fig. S1, ESI†). Whereas, its precursor or the product by hydrazine mediated cleavage, TPE-NH_2_, even though non-fluorescent in the same organic solvents, preserves an intense blue fluorescence response in water fractions above 90% manifesting the AIE behavior (Fig. S1, ESI†). The CH_3_CN–water mixture was found out as one of the better solvent systems with the highest AIE response for TPE-NH_2_ at or above 5 : 95 CH_3_CN–water making it a suitable solvent system to explore the nucleophilic attack of hydrazine to TPE-PMI and subsequent cleavage to an emissive TPE-NH_2_. Indeed, a 3% CH_3_CN in water or HEPES buffer was found as most suitable solvent system for spontaneous sensing of hydrazine. A detailed theoretical study was also carried out to understand the non-emissive character of TPE-PMI, which will be discussed later.

Next, the fluorescence behaviour of TPE-PMI in the presence of hydrazine was measured. First, as a regular practice, the fluorimetric response of 10 μM of TPE-PMI at a wide range of pH was studied in the absence and presence of one equivalent of hydrazine (Fig. S2, ESI†). It was observed that TPE-PMI remains non-fluorescence in the absence of hydrazine in the broad pH range (pH 3–12). While in the presence of hydrazine, TPE-PMI showed the highest response at a neutral pH and considerably high fluorescence response in a reasonably wide range of pH (pH 6–8), which gives us a working window in mild acidic and basic conditions with a consideration that only water or a buffer can be used for entire sensing studies. The conversion of the imide group to the corresponding amide under a strongly basic medium and protonation of the hydrazine to remain unavailable for interaction under strongly acidic conditions might be the reasons for the lower responses at higher and lower pH.

**Fig. 1:**
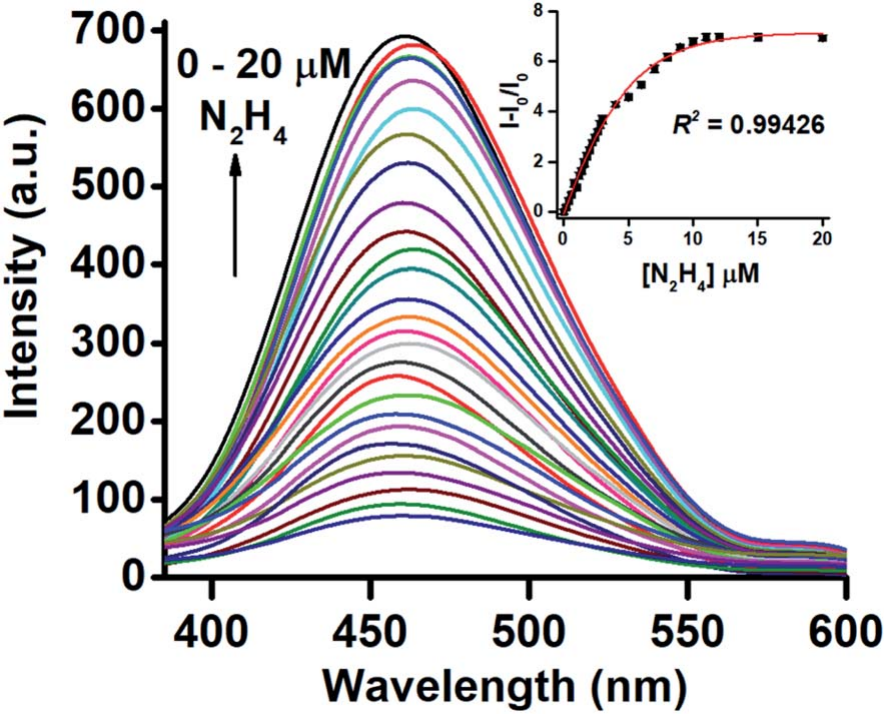
Fluorimetric response of TPE-PMI (10 μM) upon the addition of hydrazine (0 to 20 μM) in 3% CH_3_CN in HEPES [*λ*_ex_ 345 nm; *λ*_em_ 462 nm]. Inset: plot of the increment in emission against the concentration of hydrazine.

The fluorescence response was recorded by taking 10 μM of TPE-PMI in 3% CH_3_CN in HEPES (10 mM, pH 7.0) upon the incremental addition of hydrazine (0–2 equiv.) (Fig. 1). A regular increase up to ∼10 folds in fluorescence intensity of the characteristic peak of tetraphenylethylene moiety at *λ*_max_ 462 nm was observed and corresponding relative fluorescence intensities against hydrazine concentration were plotted to get a sigmoidal curve with a high *R*^2^ value (*R*^2^ = 0.99426) (Fig. 1, inset). This established the highly sensitive nature of the TPE-PMI for the detection of hydrazine in the aqueous solution.

### Selectivity studies

**Fig. 2:**
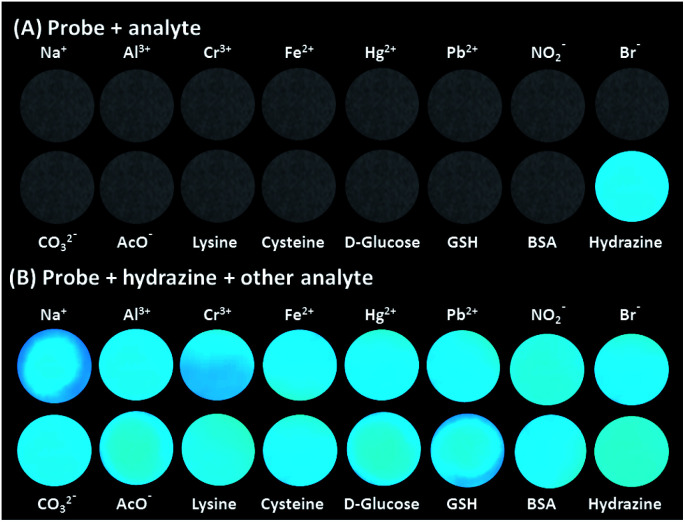
The effect of various interfering analytes on TPE-PMI drop-casted on TLC plate: (A) in the absence and (B) presence of hydrazine (viewed under long UV radiation, 365 nm).

Selectivity is the most significant parameter for assessing the performance and future applications of any analytical probe. The probe TPE-PMI, by design exhibited high selectivity towards hydrazine, and was investigated by competing it with various cations, anions which are commonly abundant in water and also with various amines, amino acids, small molecules, and reducing and oxidizing agents. The interaction of TPE-PMI with numerous prospective interfering ions like metal ions (Ag^+^, Al^3+^, Ca^2+^, Cd^2+^, Co^2+^, Cr^3+^, Cu^2+^, Fe^2+^, Fe^3+^, Hg^2+^, K^+^, Mg^2+^, Mn^2+^, Na^+^, Ni^2+^, Pb^2+^, Sn^2+^ and Zn^2+^), anions (Cl^−^, Br^−^, I^−^, NO_2_^−^, NO_3_^−^, N_3_^−^, CO_3_^2−^, HCO_3_^−^, HPO_4_^2−^, H_2_PO_4_^−^, SCN^−^, SH^−^, HSO_3_^−^, S_2_O_3_^2−^, S^2−^ and AcO^−^) 50 μM each were evaluated (Fig. S3a and b, ESI†). The study resulted in no obvious change in the fluorescence intensity for each analyte studied. Next, aliphatic and aromatic amines such as ethylenediamine, ethanolamine, triethylamine, diethylamine, ethylamine, *p*-phenylenediamine, aniline, hexamethylenetetramine (HMTA), various l-amino acids including alanine (Ala), leucine (Leu), proline (Pro), aspartic acid (Asp), glycine (Gly), cysteine (Cys), bovine serum albumin (BSA), other common chemicals like phenylhydrazine, urea, thiourea, hydroxylamine, semicarbazide were also examined against TPE-PMI. By contrast, these species caused a negligible output of the fluorescence intensity from their respective solutions (Fig. S3c, ESI†). Further, reductants like glutathione (GSH), glucose (Glu), NaBH_4_, HQ, and oxidants like BQ, H_2_O_2_, sodium borate, sodium hypochlorite, 50 μM each were evaluated (Fig. S3c, ESI†). It was found that the fluorescence intensity of TPE-PMI was highly enhanced only by the addition of hydrazine. The other redox compounds did not cause any significant changes in the fluorescence intensity. All the above results indicated that TPE-PMI undergoes a typical nucleophilic attack of hydrazine proposed for the Gabriel synthesis pathway with excellent selectivity toward hydrazine in aqueous media. In a separate study, we evaluated the specificity of TPE-PMI towards hydrazine on solid-phase with selected analytes on TLC plates. In Fig. 2a, TLC based detection showed that only hydrazine results in an obvious fluorescence response while the competitors, even the reductive species (cysteine, d-glucose and GSH), did not cause any sign of fluorescence in the solid phase. Then we conducted the detection of hydrazine in the presence of all of selected interference molecules (Fig. 2b). The results show that there is no obvious difference in fluorescence intensity. This study indicated the high selectivity of TPE-PMI towards detecting hydrazine on the solid phase as well.

### DFT studies

**Fig. 3:**
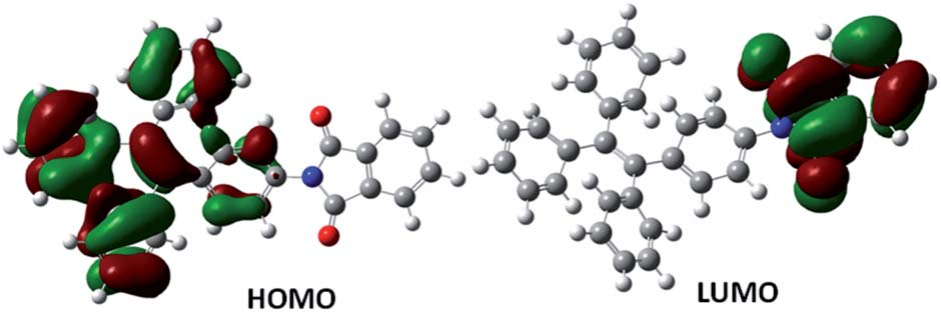
Frontier molecular orbital diagram of HOMO and LUMO of TPE-PMI in ground state geometry.

To understand the electronic behaviour of TPE-PMI and its cleaved form after interaction with hydrazine (*i.e.*, TPE-NH_2_) quantum mechanical studies were carried out at the density functional theory (DFT)^52^ and time-dependent density functional theory (TD-DFT)^53^ levels using the Gaussian 09 program to understand the ground and excited state behaviour of TPE-NH_2_ and TPE-PMI. Becke's three-parameter exchange functions (B3)^54^ with Lee–Yang–Parr correlation^55^ function (LYP) were employed using 6-311G basis set for the geometry optimization calculations. The probe TPE-PMI showed non-fluorescent nature both in the solution as well as in the solid-state. Fig. 3 showed that a major portion of the π-cloud is shifted from HOMO of the TPE-moiety and exists near to the phthalimide moiety in the LUMO. During the excitation from HOMO to LUMO, the disappearance of π-electron cloud from the central ethylene moiety as well as from three of the four phenyl rings of tetraphenylethylene was observed (Fig. 3). During S_0_–S_1_ transition, the fourth phenyl ring of the TPE moiety, which is directly connected to the electron-withdrawing phthalimide part and located on the other side of the bond, can create a channel for shifting of the π-electron cloud because of their coplanarity. Therefore, the non-fluorescent nature of TPE-PMI could be correlated to the disappearance of π electron cloud from the major part of the TPE moiety in the course of HOMO to LUMO transition. This intramolecular charge transfer (ICT) process is successively followed by a non-radiative decay from this charge-separated excited state. The optimized singlet excited state of this species is more or less having a similar geometry as the ground state (Fig. S4, ESI†). The energy gap between the excited state at its optimized geometry and the ground state at this geometry was found to be very small (∼0.73 eV). As per the energy gap law,^63^ this shows a chance of non-radiative decay of the singlet excited state. Additionally, the aggregated state is also not anticipated to have a better π-delocalization (even though it becomes entirely planar) as the electron-withdrawing part of the molecule will still inhibit flow of π-electronic cloud over the TPE part. Whereas, for TPE-NH_2_ the S_0_–S_1_ transition arising due to the HOMO → LUMO excitation was characterized by an association of the π-cloud from the aniline moiety towards the central ethylenic part (Fig. S5 and S6, ESI†).^44^ A better co-planarity resulting in from improved delocalization can be expected in the aggregated form and might be the reason behind the fluorescent nature of TPE-NH_2_ in this state.

### The mechanistic aspect of hydrazine sensing by TPE-PMI

**Scheme 1:**
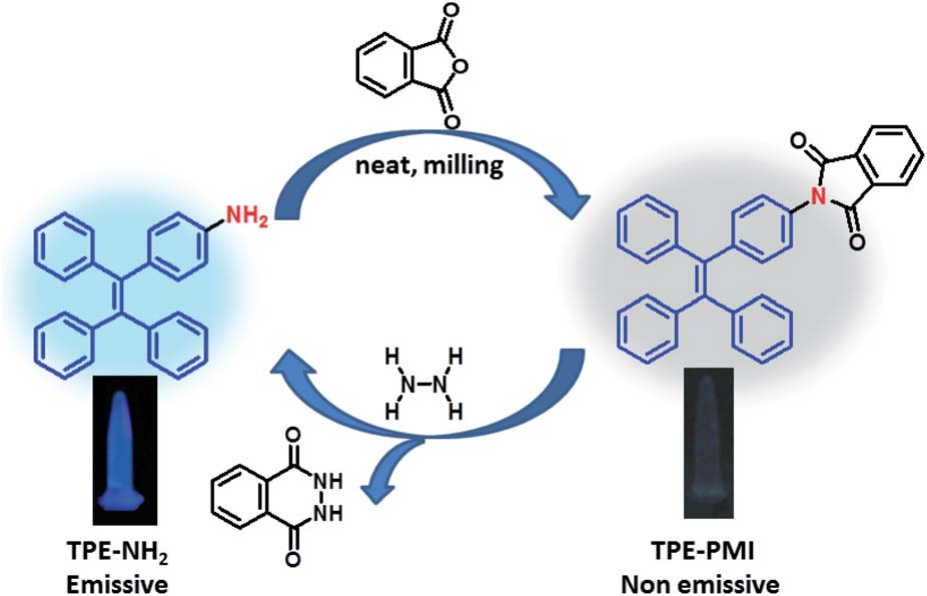
Synthesis and sensing mechanism of TPE-phthalimide (TPE-PMI).

Based on the previous report,^44^TPE-NH_2_ is considered as the fluorescence signaling unit, and the quenching of fluorescence in TPE-PMI is predicted *via* the ICT process from the TPE fluorophore to the phthalimide moiety. However, in an appropriate solvent system hydrazine converts TPE-PMI to the corresponding free amine, *i.e.* AIE-active TPE-NH_2_*via* “Gabriel synthesis” pathway in two steps. The nucleophilic addition of hydrazine to the carbonyl carbon, in the first step, is followed by the subsequent nucleophile attack on the carbonyl group would induce the release of phthalhydrazide and the TPE-NH_2_ would be generated (Scheme 1). In order to verify the proposed mechanism, further studies were conducted. First, the LC-MS of sensing solution was recorded after addition of 0.5 equiv. of hydrazine. At different retention time, the peak at *m*/*z* 478 [M + H]^+^ representing TPE-PMI and at *m*/*z* 348 [M + H]^+^ corresponding to the TPE-NH_2_ were observed clearly indicating hydrazine mediated conversion of TPE-PMI to TPE-NH_2_ (Fig. S7, ESI†). Next, to determine the structure of the generated byproducts, a reaction was kept with TPE-PMI (30 mg) and hydrazine in 1 : 1 CH_3_CN–water and monitored by TLC. The complete conversion of TPE-PMI was observed after 15 min of stirring at room temperature and the product spot appeared at the same *R*_f_ of TPE-NH_2_. The product was isolated and characterized by ^1^H NMR spectra which matched well with TPE-NH_2_. Thus, the proposed mechanism of cleavage of phthalimide unit of TPE-PMI by hydrazine to generate AIE-active TPE-NH_2_ by “Gabriel synthesis” pathway is well-established.

### Limit of detection for hydrazine

One of the important characteristics that prove the effectiveness of a probe is its limit of detection (LOD). The performance of the probe (TPE-PMI) as an analytical tool towards quantitative detection of hydrazine was evaluated under optimized conditions by plotting relative fluorescence intensities against the concentration of hydrazine at a lower concentration range (Fig. S8, ESI†). A good linear correlation (*R*^2^ = 0.99803) was observed even at the micromolar concentration range (0.2–3 μM), from which the limit of detection was considered as 0.2 μM (or 6.4 ppb) as the lowest detectable value. This established the highly sensitive nature of the TPE-PMI for the detection of hydrazine in the aqueous solution.

### Determination of hydrazine in real samples

**Fig. 4:**
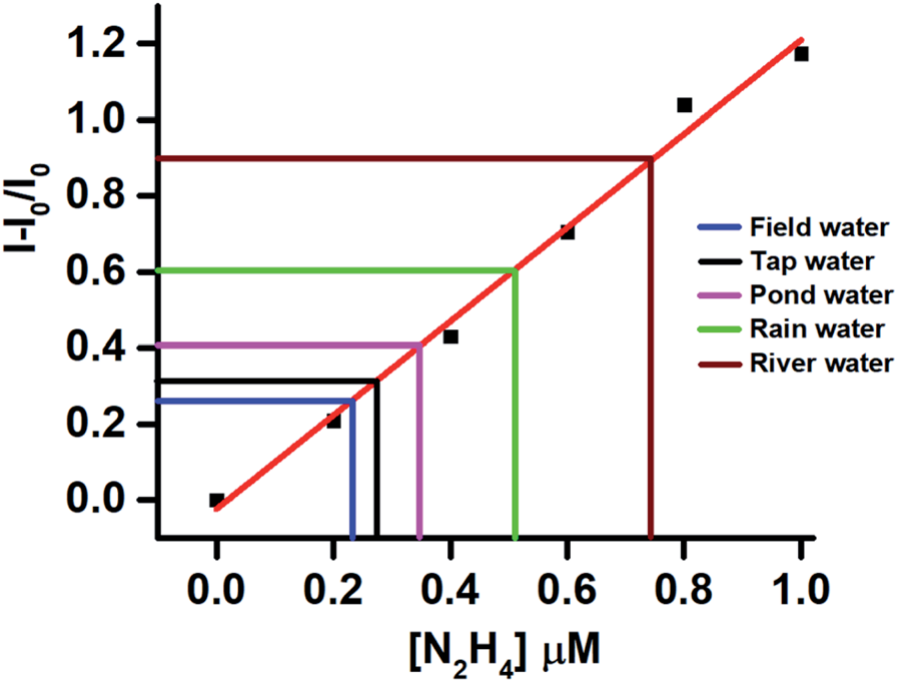
Fluorescence response of TPE-PMI towards real samples spiked with a measured amount of hydrazine on a standard plot of relative fluorescence intensities of TPE-PMI against the concentration of hydrazine (0.2–1.0 μM).

The real sample analysis was conducted to monitor the hydrazine contamination in different water samples like field, tap, pond, rain, and river water. The water samples were collected from the above water bodies and were spiked with different concentrations of hydrazine. The fluorescence responses of the aqueous solutions were recorded, and the intensity at 462 nm was plotted onto the standard graph (Fig. 4). In general, all the real samples showed good recovery of hydrazine between 92 to 97% (Table S1, ESI†). The little lower recovery in water samples may be because of the presence of some competing species.

### Demonstration of “on-site” detection of hydrazine

**Fig. 5:**
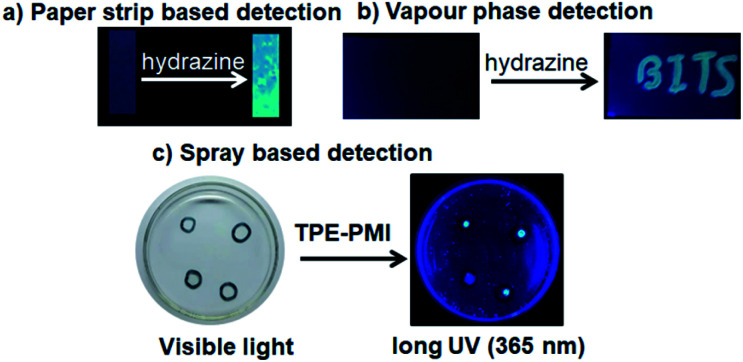
Images of detection of hydrazine in (a) paper strips dipped in TPE-PMI (1 mM) before and after exposure to hydrazine; (b) vapour phase detection by dipping TLC plate in TPE-PMI (1 mM) solution and exposing it to hydrazine vapour; (c) in solution phase by drop casting different concentrations of hydrazine on the culture dish and spraying TPE-PMI (100 μM) on it; images captured in bright field (left) and under UV light (365 nm).

After realizing the highly selective nature of TPE-PMI towards hydrazine, we validated the applicability of the said probe on the paper strip and TLC plate to make it more acceptable for practical applications. For this purpose the filter paper was initially dipped in 1 mM solution of TPE-PMI in CH_3_CN and dried under vacuum at room temperature. The TPE-PMI soaked paper did not show any fluorescence under UV light but started giving blue fluorescence immediately upon exposure to hydrazine solution (10 μM) (Fig. 5a). Further, a similar test was performed on TLC plate to demonstrate detection of hydrazine in the vapour state. The TLC plate was engraved as “BITS” with TPE-PMI solution and exposed to hydrazine vapour. The non-fluorescent TLC plate immediately started showing blue fluorescence (Fig. 5b). To assess the sensing ability of TPE-PMI towards the real-world applications in the solution phase, a probe solution in CH_3_CN was prepared and kept in a spraying bottle. For the demonstration purpose, a small drop of the aqueous solution of hydrazine solutions (5 μM, 10 μM, 50 μM, 100 μM) was placed in a Petri dish and TPE-PMI solution (100 μM) in CH_3_CN was sprayed. The immediate appearance of blue fluorescence was observed from the drop under the long UV light (365 nm) (Fig. 5c). Thus, this easy user-friendly procedure can offer a useful approach for onsite analysis and detection of hydrazine.

**Fig. 6:**
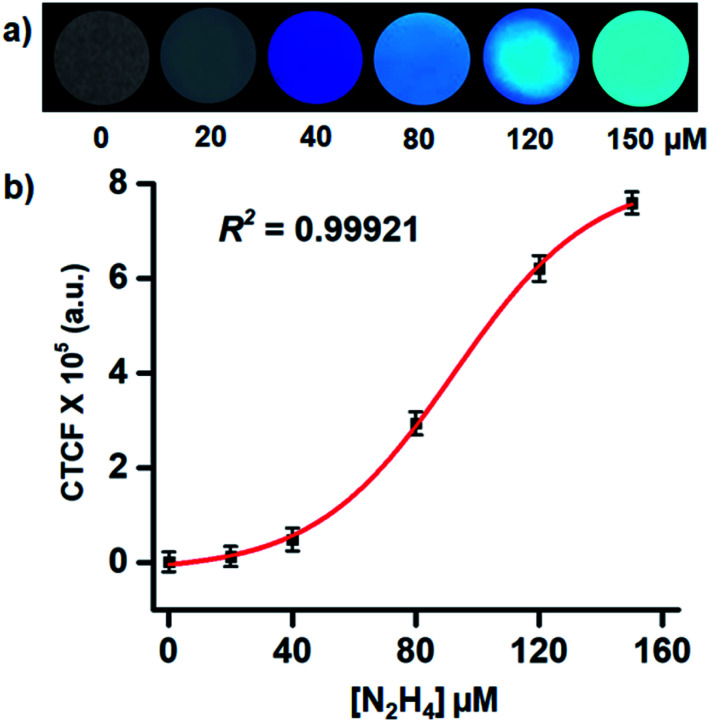
(a) The images of TLC plates drop-casted by TPE-PMI and dipped in different concentrations of hydrazine; (b) plots of the CTCF values of the images of TLC plates captured on a smartphone and processed using ImageJ analysis software.

Recently, smartphone based devices are successfully employed in the detection and quantitation of analytes by a quick analysis of images and is slowly becoming a user-friendly approach for on-site detection.^64^ After successful demonstration of the solid phase detection of hydrazine, we felt, it will be worth carrying out image analysis for the quantitation of hydrazine in the solid phase. Again, we utilized TLC plates as the solid platform. The TLC strips were prepared by drop-casting the probe solution (1 mM in CH_3_CN) on the TLC plate and dipping them in the aqueous solution of the different concentrations of hydrazine. In all the cases the TLC plates showed blue fluorescence of different intensities when placed under UV lamp (365 nm). The images were captured with the camera and subjected to ImageJ analysis. Clearly, the observed fluorescence became brighter with exposure to a higher concentration of hydrazine. For the analysis of each sample, four different regions were selected randomly within the circular region using the background as a control and corrected total cell fluorescence (CTCF) value of the images were plotted against the concentrations of hydrazine. More importantly, the solid phase provides a sufficient fluorescence response from a 40 μM of hydrazine with a regression coefficient of *R*^2^ = 0.99921 (Fig. 6). This shows the scope of using the image analysis technique for the possible quantification of hydrazine for real-life applications.

### Direct detection of hydrazine in soil samples

**Fig. 7:**
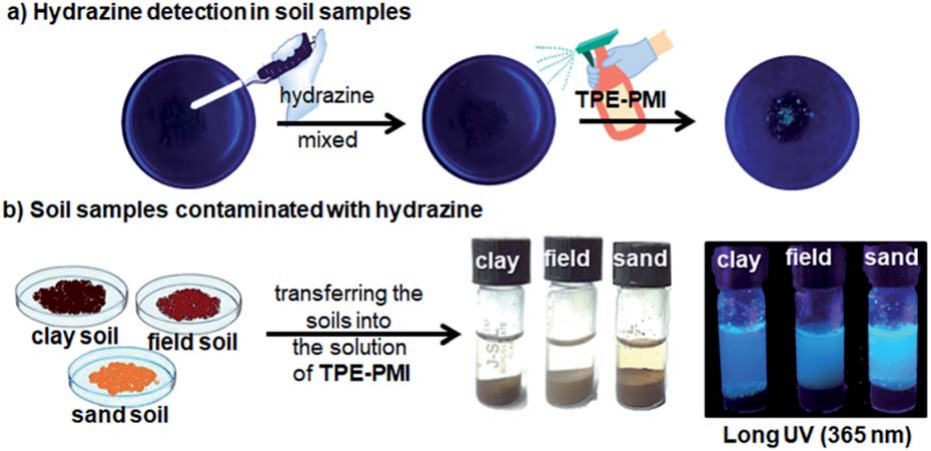
On-site detection of hydrazine: (a) in solid-phase the hydrazine contaminated soil was sprayed with TPE-PMI (1 mM) and viewed under UV light; (b) different soil types like clay, field and sand soil were exposed to hydrazine (1 mM) and transferred into the solution contained TPE-PMI (1 mM) and the solutions were viewed under UV light (365 nm).

To extend the applicability of this approach for the possible detection of hydrazine in environmental soil samples, in a qualitative way, some amount of the field soil was taken in a watch glass and contaminated with hydrazine followed by spraying TPE-PMI (1 mM) solution before capturing images. The blue fluorescence was observed from the soil sample under long UV light (365 nm) (Fig. 7a).

The initial success encouraged us to further investigate the direct detection of hydrazine in various soil samples (clay soil, field soil, and sand soil). Initially, the soil (1 g) was contaminated with the known concentration of aqueous hydrazine (50 μL of 1 mM). Next, the contaminated soils were added to the solution of TPE-PMI (100 μM, in 3% CH_3_CN in HEPES) and the fluorescence change was monitored. A distinct blueish emission was observed within a minute in all the soil samples (Fig. 7b). The fluorescence signals from these solutions were visible even after 10 days. Next, in the same way like solid phase based image analysis, the quantitation of hydrazine in the sand sample was carried out through image analysis. The images of variable amounts of hydrazine contaminated sand transferred to the solution of TPE-PMI were captured by a 64 megapixels mobile camera and processed through Image-J analysis software. A good correlation plot between CTCF and the amount of hydrazine with high regression coefficient (*R*^2^ = 0.98494) was obtained from 0.05 μmoles to 0.5 μmoles (Fig. S9, ESI†). These studies clearly demonstrate the potential real-world applicability of TPE-PMI for the detection and quantitation of hydrazine.

### Detection of hydrazine in live cells

To further explore the usefulness of TPE-PMI in the living species, we carried out *in vitro* studies for the detection of hydrazine in HeLa cells. In this regard, the cytotoxicity of TPE-PMI by the MTT assay at different concentrations was determined (Fig. S10, ESI†). Three cell lines namely A549, HEPG2 and HeLa cells were selected for cell viability study. As seen from the bar diagram, no significant cell death of any of the cell lines was observed up to 50 μM of TPE-PMI making it safe to use on human cell lines even at reasonably higher concentrations. The cytotoxicity study allowed us to use TPE-PMI for *in vitro* sensing in HeLa cells. For this, cells were pre-treated with a large excess of hydrazine (50 μM) for 30 min, followed by incubation with TPE-PMI (10 μM). This followed a washing step to remove any loose probe molecules that are present in the culture media. Initially, a time-dependent fluorescence response was carried out (Fig. S11, ESI†). The fluorescence turn-on response in the presence of hydrazine at different time intervals (10 min, 15 min, 30 min) was imaged upon incubation with TPE-PMI. It was observed that as time increases, the HeLa cells show enhanced fluorescence from the intracellular region. It indicates that probe TPE-PMI is suitable for the *in vitro* sensing of hydrazine; can easily penetrate the cell membrane to enter into the living cells and interact with hydrazine present in the cytosol and trigger a fluorescence response. The highest manifestation of fluorescence was observed after 30 min which was considered as the ideal time for the hydrazine to enter into the live cells. A similar study was replicated by pre-treatment of HeLa cells with various concentrations of hydrazine (0–100 μM) (Fig. S12, ESI†). Once again, the blue fluorescence was observed even if the cells were exposed to only 10 μM of hydrazine. Thus, the probe can be used for *in vitro* detection and wash-free imaging of hydrazine *via* cell imaging even at a reasonably low concentration level.

### Comparison with available AIE-based probes

**Fig. 8:**
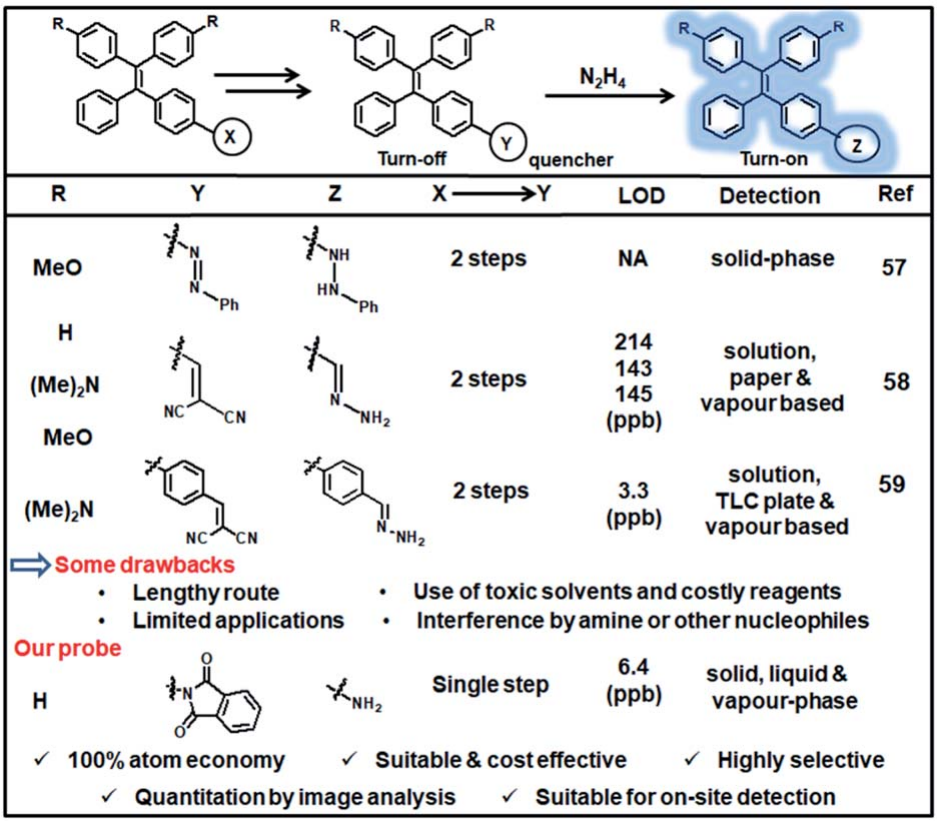
Comparison table of previously reported TPE-based probes towards hydrazine detection.

In a separate analysis, the available AIE-based probes for hydrazine detection with respect to synthetic strategy, cost-effectiveness, selectivity, detection limit, application scope *etc.* were compared (Fig. 8 and Table S2, ESI†). It can be observed that the present sensing platform is advantageous in many aspects like cost-effective one-step atom-efficient synthesis, high selectivity, low LOD *etc.* Notably, the preparation cost of the present probe is 26 times lower than the TPE-azide probe reported by Liu's group;^57^ as estimated from the known TPE derivative and using Sigma-Aldrich Chemicals for cost calculations. Moreover, the probe was validated for on-site detections of hydrazine by a smartphone-based image capturing and quantitation by ImageJ analysis.

## Conclusions

In summary, a new AIE-based fluorimetric probe (TPE-PMI) has been successfully utilized for selective detection of hydrazine in solid, liquid and vapour phases. TPE-PMI was synthesized in a one-step solvent-free mechanochemical approach by the condensation of AIE-active TPE-NH_2_ with phthalic anhydride to afford the probe which is non-emissive both in solution and solid phase by the presence of phthalimide group as AIE-quencher. TPE-PMI detects hydrazine by the unique Gabriel synthesis pathway leading to the cleavage of phthalimide unit to release the precursor TPE-NH_2_, which emits blue fluorescence. The reported detection tool holds a linear relationship with hydrazine in a wide range of concentrations. The LOD was determined as 6.4 ppb for hydrazine which is lower than the threshold limit set by US-EPA (10 ppb). Its high selectivity was established by interfering with several metal anions, anions, amines, amino acids, and even common oxidants and reductants. Further, the utility of this sensing tool was demonstrated in real-sample analysis, and quantitation was done in various environmental water and soil samples. Moreover, the detection of hydrazine was successfully demonstrated on solid platforms like TLC plates and paper strips. Finally, TPE-PMI was found effective in the detection and wash-free imaging of hydrazine *in vitro* in live cells (HeLa). Overall, the probe is simple, cost-effective and highly selective to hydrazine by design, and environmentally benign and atom-efficient by synthetic strategy. The smartphone-based image capturing and quantitation by ImageJ analysis is an additional merit of the current sensing system to consider it as an efficient “on-site” detection tool for the future.

## Author contributions

Analytical studies including solid-phase studies, imaging S. D. H.; and A. A. B; analytical studies including selectivity studies, real sample analysis *etc.* R. U. G.; synthesis of AIE probe and spectral analysis, D. D. and V. G. N.; soil sample analysis and imaging A. A. B.; discussion, review: R. A.; *in vitro* hydrazine detection and cytotoxicity studies, V. P. M. and K. K. M.; implementation of experiments, writing and reviewing M. B.; inception of the concept and management of the whole project, A. C.

## Conflicts of interest

There are no conflicts to declare.

## Supplementary Material

RA-011-D1RA03563K-s001
