# Supplementary material for: Phthalimide conjugation turns the AIE-active tetraphenylethylene unit non-emissive: its use in turn-on sensing of hydrazine in solution and the solid- and vapour-phase
Source: RSC Adv. 2021 Jun 15;11(35):21269–78. doi: 10.1039/d1ra03563k (PMC9034109; doi:10.1039/d1ra03563k)
Supplement: RA-011-D1RA03563K-s001 [file RA-011-D1RA03563K-s001.pdf]

Electronic Supplementary Material (ESI) for RSC Advances.
This journal is © The Royal Society of Chemistry 2021

Supporting Information

Phthalimide Conjugation Turns AIE-Active Tetraphenylethylene Unit Non-Emissive: Its use in Turn-on Sensing of Hydrazine in Solution, Solid- and Vapour-Phase

Sharanabasava D. Hiremath,[a†], Ram U. Gawas [a†] Dharmendra Das,[a] Viraj G. Naik,[a] Akhil A. Bhosle,[a] Vishnu Priya Murali,[b] Kaustabh Kumar Maiti,[b] Raghunath Acharya,[c,d] Mainak Banerjee,*[,a] Amrita Chatterjee*[,a]

[a]Department of Chemistry, BITS, Pilani- K. K. Birla Goa Campus, NH 17B Bypass Road, Zuarinagar, Goa 403726, India.
[b]CSIR-National Institute for Interdisciplinary Science and Technology (CSIR-NIIST), Thiruvananthapuram, Kerala 695019, India.
[c]Radiochemistry Division, Bhabha Atomic Research Centre, Trombay, Mumbai 400085, INDIA.
[d]Department of Atomic Energy, Homi Bhabha National Institute, Mumbai 400094, INDIA.
Corresponding authors e-mail: amrita@goa.bits-pilani.ac.in (AC); mainak@goa.bits-pilani.ac.in (MB)

Table of Contents

Sr. No.	Contents	Pg. No.
1	Synthesis of **TPE-NH$_2$**	S2
2	Fluorimetric responses of **TPE-PMI** and **TPE-NH$_2$** in various solvents	S2
3	pH study of **TPE-PMI** with and without hydrazine	S3
4	Selectivity study of **TPE-PMI** towards hydrazine	S3
5	Quantum mechanical studies of **TPE-PMI** and **TPE-NH$_2$**	S4-S5
6	LCMS analysis of the sensing solution	S5
7	Limit of detection	S6
8	Detection of hydrazine in various real samples in solution phase	S6
9	Detection of hydrazine in sand samples	S7
10	Cytotoxicity and detection of hydrazine in HeLa cells	S7-S8
11	Comparison of the present study and previous reports for the detection of hydrazine	S9-S11
12	References	S11
13	^{1}H, ^{13}C NMR, HRMS and IR spectra of **TPE-PMI** and **TPE-NH$_2$**	S13-S15

1. Synthesis of 4-(1,2,2-triphenylvinyl)benzenamine (TPE-NH_2)[1]

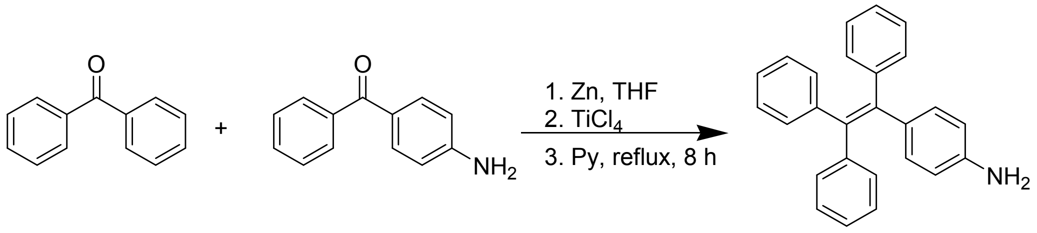

In a three-necked flask, zinc powder (0.8 g, 12 mmol) in 20 mL anhydrous THF was taken and the reaction vessel was kept under nitrogen atmosphere. The mixture was cooled to 0 °C and $TiCl_4$ (0.65 mL, 6 mmol) was slowly added by a syringe. The suspension was warmed to room temperature and stirred for 30 min, then heated at reflux for 2.5 h. The mixture was again cooled to 0 °C, charged with pyridine (0.25 mL, 3 mmol) and stirred for 10 min at the cold condition. An equimolar mixture of 4-aminobezophenone (475 mg, 2.4 mmol) and benzophenone (440 mg, 2.4 mmol) in 20 mL of THF was added slowly. After complete addition, the reaction mixture was heated at reflux for 8 h. The reaction was quenched by addition of 10% aqueous K_2CO_3 solution and extracted with CH_2Cl_2. The organic layer was collected and concentrated. The crude product was purified by column chromatography to give the desired product, **TPE-NH_2** (320 mg, yield: 72%) as yellow solid. ^{1}H NMR (300 MHz, $CDCl_3$): δ (ppm) 3.58 (2H, s, exchangeable), 6.46 (2H, d, J = 8.0 Hz), 6.89 (2H, d, J = 8.0 Hz), 7.03-7.26 (15H, m). ^{13}C NMR (75 MHz, $CDCl_3$), δ (ppm) 114.4, 126.2, 126.3, 127.6, 127.8, 131.45, 131.49, 131.6, 132.6, 134.0, 139.4, 141.1, 144.27, 144.30, 144.5, 144.9; ESI-MS: m/z 348 $[M + H]^+$.

2. Fluorimetric responses of TPE-PMI and TPE-NH_2 in various solvents

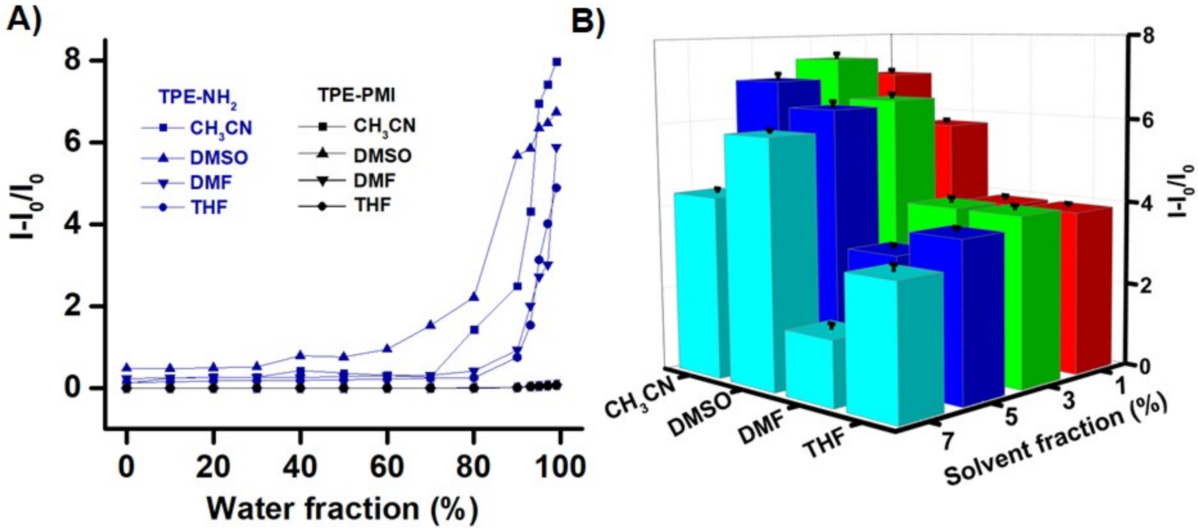

Fig. S1. A) Change in the fluorescence intensity of **TPE-NH_2** and **TPE-PMI** at 492 nm as a function of different percentages of the water fraction in solvents like CH_3CN, DMF, DMSO and THF; B) The fluorescence response of **TPE-PMI** towards hydrazine in different solvent fractions.

3. **pH study of TPE-PMI with and without hydrazine**

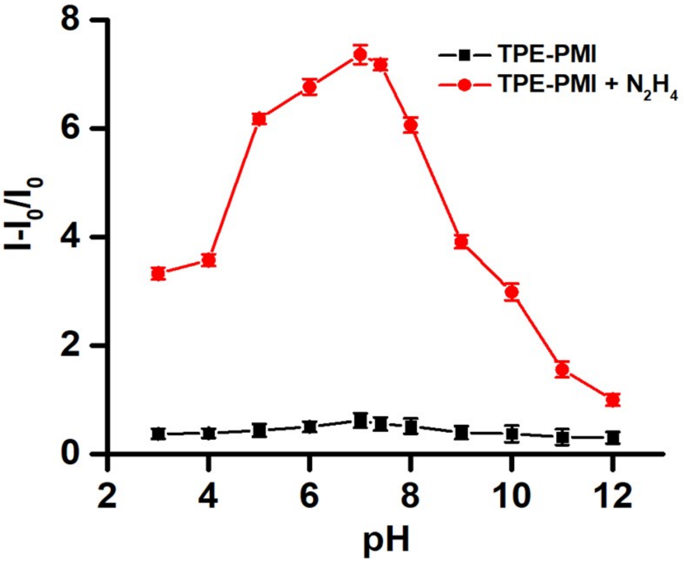

Fig. S2 Fluorescence intensity change of **TPE-PMI** (10 μM) in the absence and presence of hydrazine (10 μM) in 3% CH_3CN/ buffer solution [pH 2-6 acetate buffer, pH 7-8 HEPES buffer and pH 9-12 carbonate buffer] (λ_{ex} = 345 nm, λ_{em} = 462 nm).

4. **Selectivity study of TPE-PMI towards hydrazine**

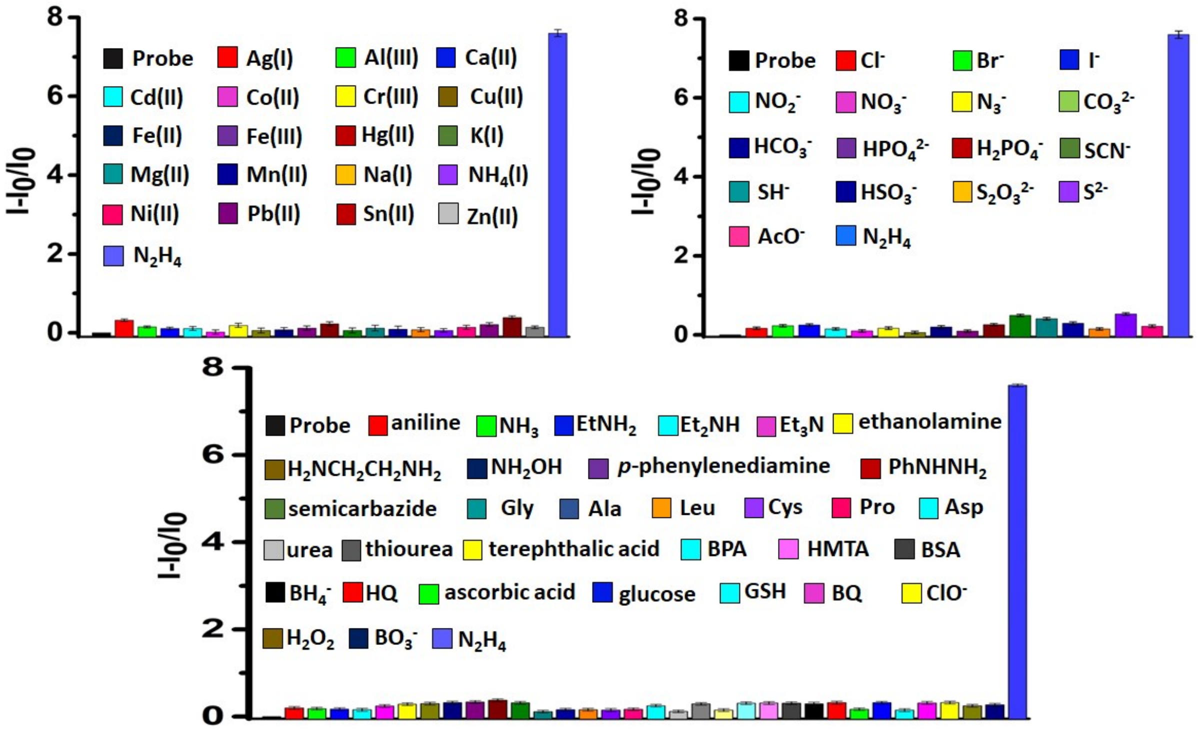

Figure S3. The fluorimetric responses of various metal ions (50 μM), anions (50 μM) and other molecules (50 μM) towards **TPE-PMI** (10 μM) under optimized conditions (λ_{ex} 345 nm; λ_{em} 462 nm).

5. Quantum mechanical studies of TPE-PMI and TPE-NH_2

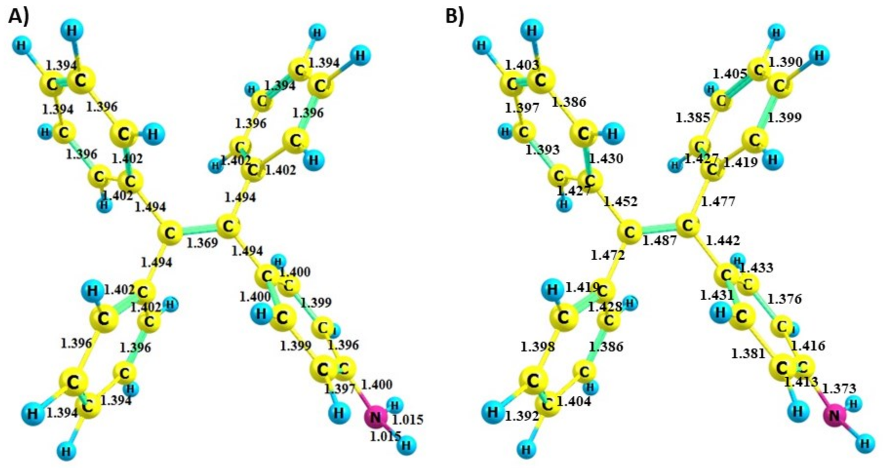

Fig. S4 (A) Ground state geometry (optimized at the B3LYP/6-311G level) and (B) excited state geometry (optimized at the Td-B3LYP/6-311G level) of **TPE-NH_2**.

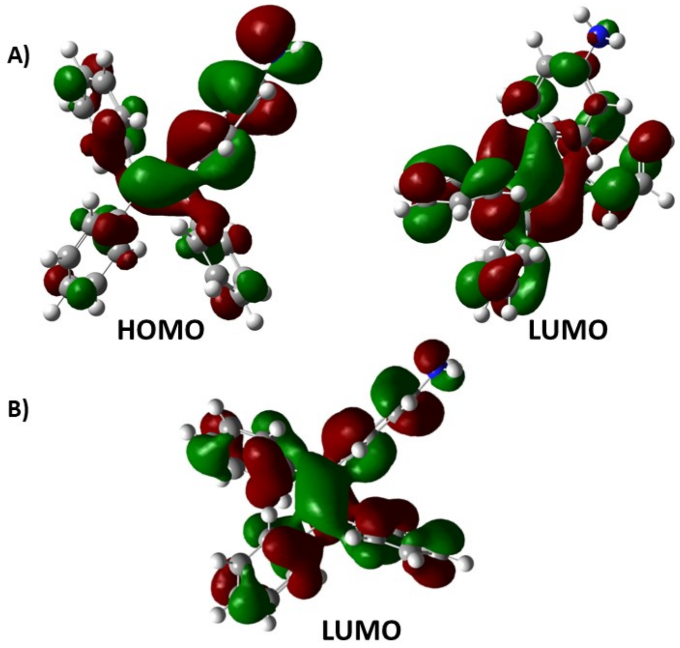

Figure S5. The frontier molecular orbitals of **TPE-NH_2** at the optimized (A) ground state and (B) excited state geometries.

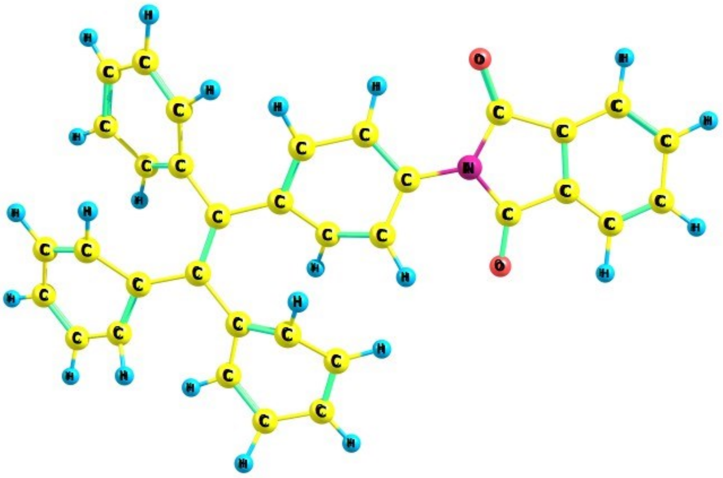

Figure S6. Ground state geometry (optimized at the B3LYP/6-311G level) of **TPE-PMI.**

6. LCMS analysis of the sensing solution

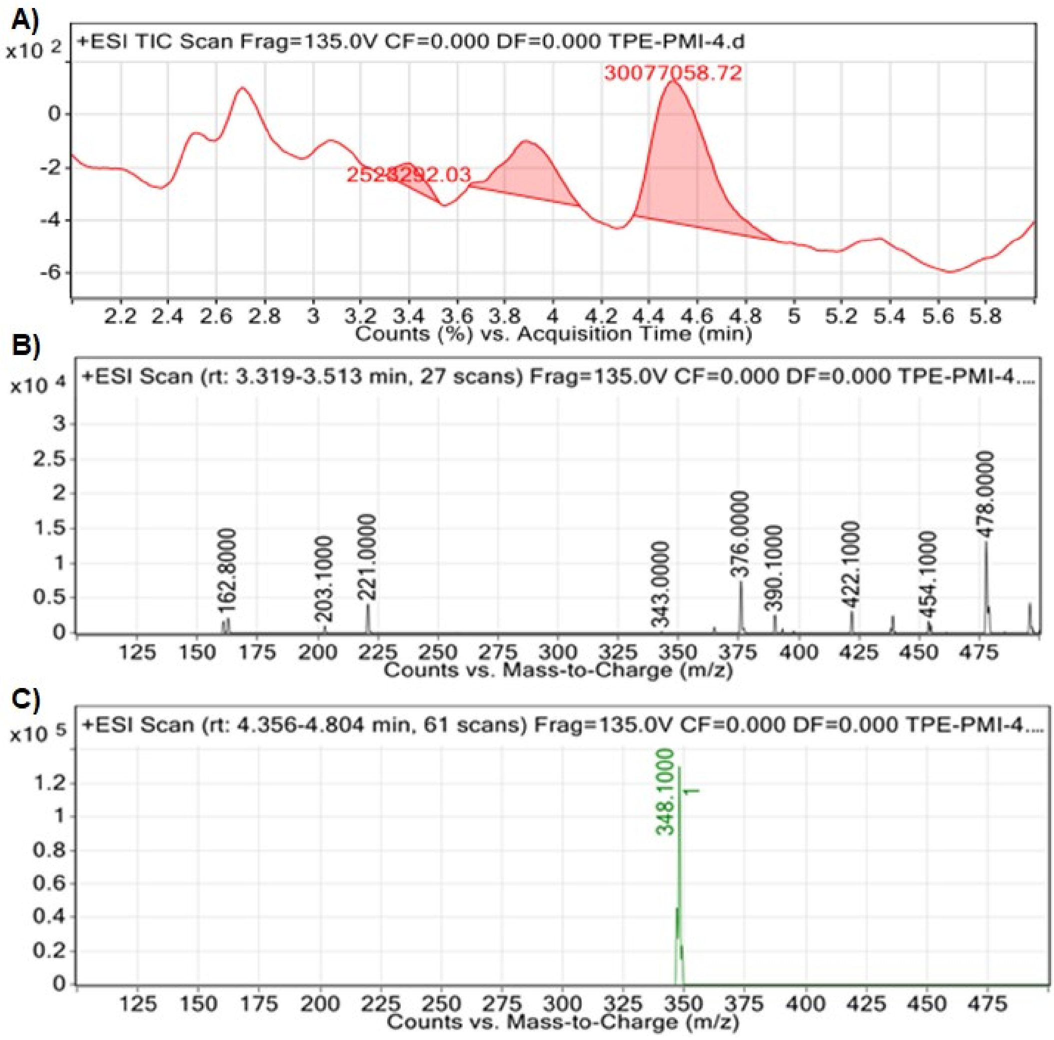

Fig. S7 (A) LCMS analysis of the sensing solution (B) showing the cleavage of **TPE-PMI** (*m/z* peak at 478 $[M + H]^{+}$); (C) upon addition of hydrazine to form a new *m/z* peak at 348 which corresponds to M + H of **TPE-NH$_2$**.

7. Limit of detection (LOD)

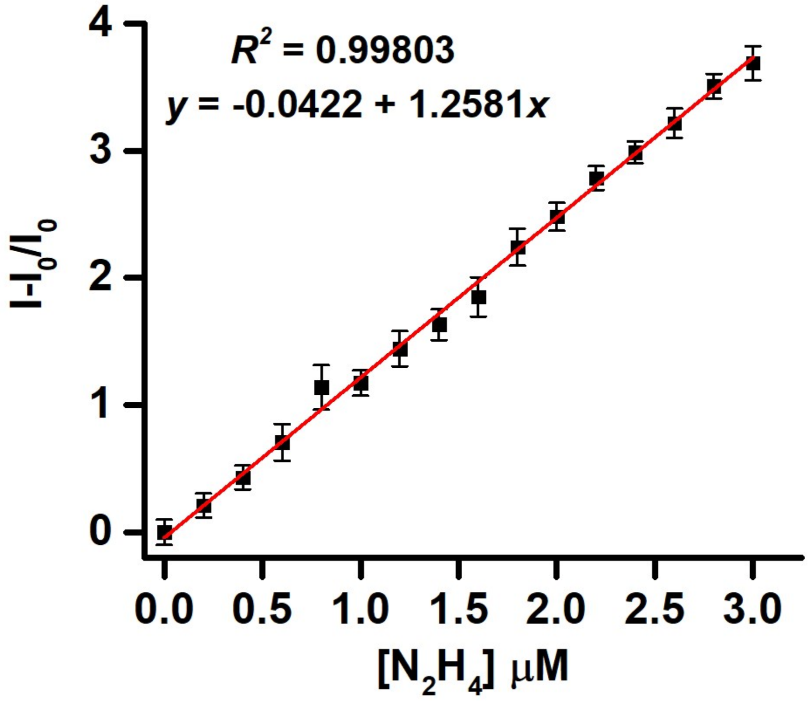

Fig. S8 Plot of relative intensities vs concentration of hydrazine showing an excellent linear fit (R^2 = 0.99803) which ensures that **TPE-PMI** can detect hydrazine as low as 0.2 μM (or 6.4 ppb) (λ_{ex} 345 nm, λ_{em} 462 nm).

8. Detection of hydrazine in various real samples in solution phase

The concentration of hydrazine in five different water samples was closely matched with the amount of hydrazine spiked showing a recovery of 92-97%.

Table S1. Real sample analysis for hydrazine

Sr. No.	Sample	Concentration found from the graph (μM)	Actual concentration (μM)	Recovery (%)	RSD (%) (n = 3)
1.	Field water	0.24	0.25	96	4.1
2.	Tap water	0.28	0.30	93.3	3.3
3.	Pond water	0.33	0.35	94.2	3.6
4.	Rain water	0.53	0.55	96.3	3.9
5.	River water	0.74	0.8	92.5	3.3

9. Detection of hydrazine in sand samples

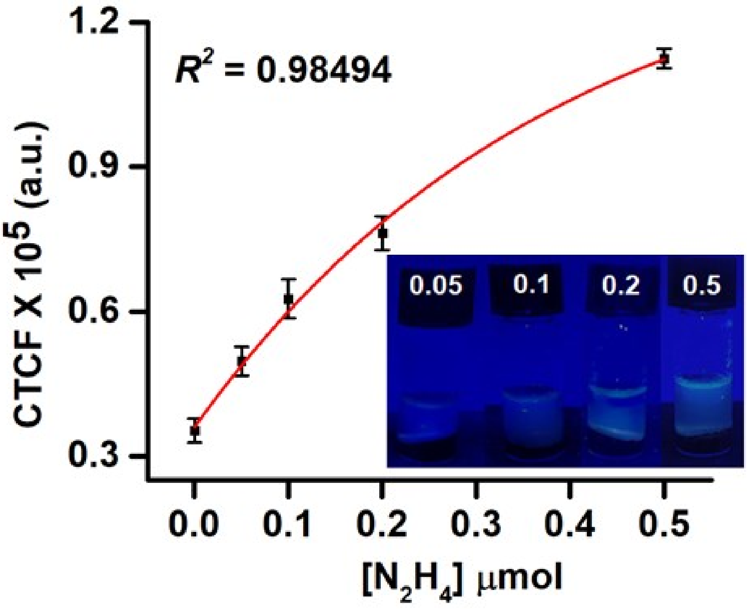

Fig. S9. The plots of the CTCF values of the images of the sensing assay at different concentrations of hydrazine spiked on the sand samples under long UV-light, captured on a smartphone and processed using ImageJ analysis software.

10. Cytotoxicity and detection of hydrazine in HeLa cells

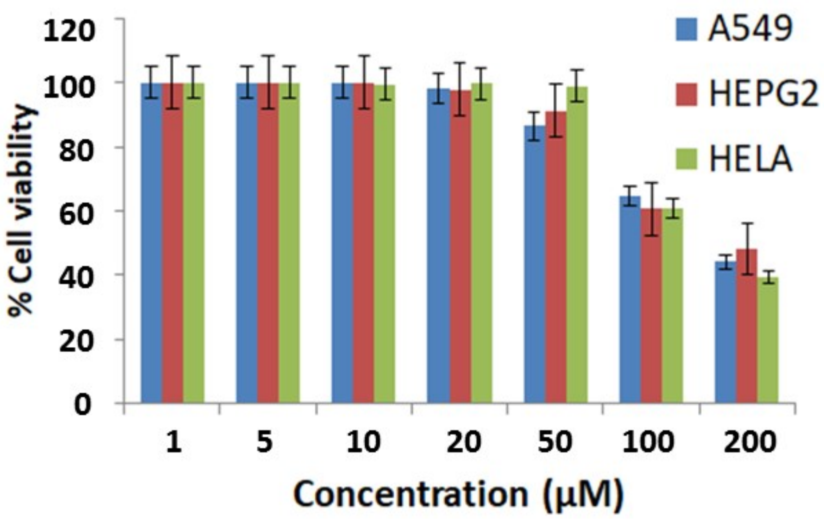

Fig. S10 Cytotoxicity by MTT assay of **TPE-PMI** at different concentrations is presented above. The bar graphs showed that more than 60% of the total cells were still viable in the presence of up to 50 μM of **TPE-PMI** in the culture media of HeLa cells after 24 h. This implies that the **TPE-PMI** is nontoxic in nature.

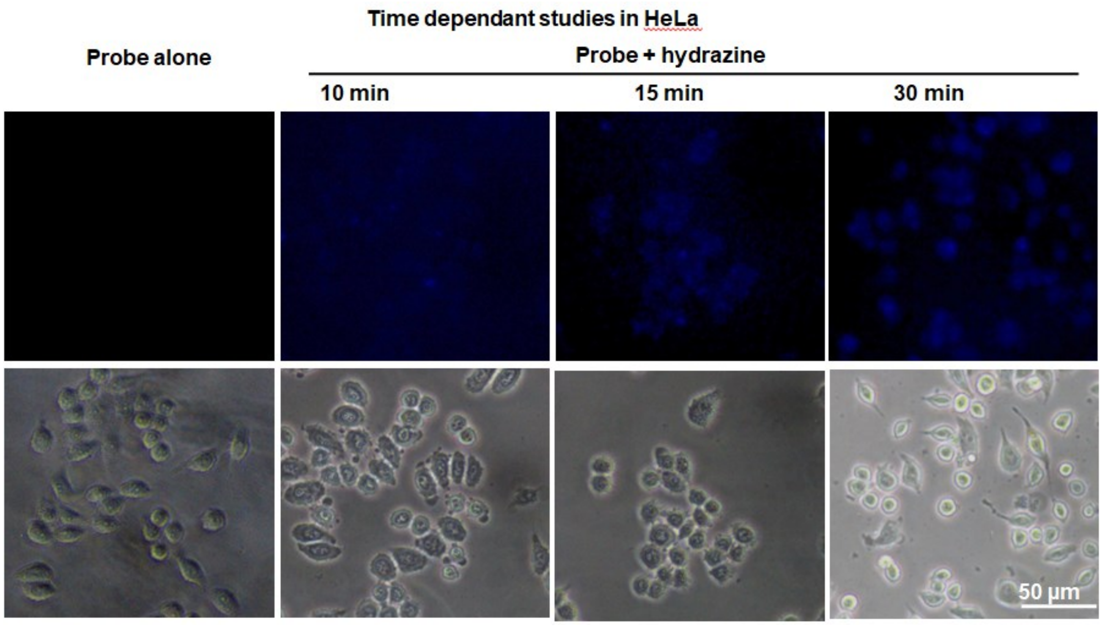

Fig. S11 Time-dependent fluorescence detection of toxic hydrazine deposition in HeLa cells treated 50 μM hydrazine in serum-free DMEM to utilized sensing fluorescence probe **TPE-PMI**. HeLa cells incubated for 10-30 mins and washed with PBS buffer. Next, the cells were treated with **TPE-PMI** (10 μM) for 60 min followed by PBS wash (3 times) and imaged under DAPI filter of Nikon fluorescent microscope.

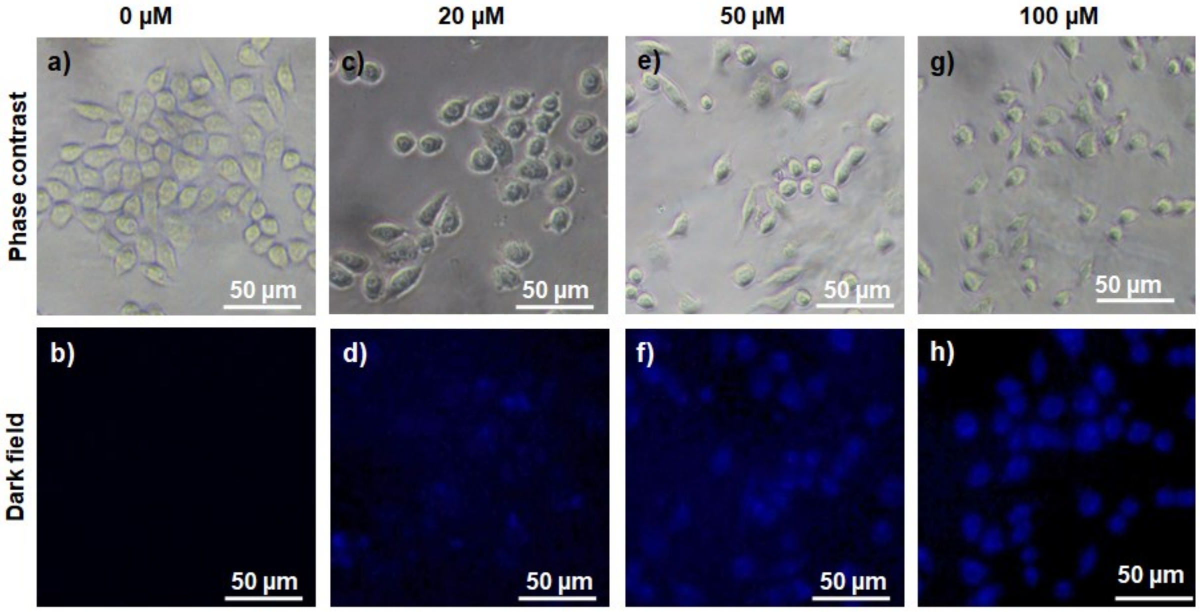

Figure S12. Pretreatment of HeLa cells with varying concentrations of hydrazine (a) and (b) **TPE-PMI** without hydrazine; (c) and (d) **TPE-PMI** and 20 μM hydrazine; (e) and (f) **TPE-PMI** and 50 μM hydrazine; (g) and (h) **TPE-PMI** and 100 μM hydrazine.

11. Table S2 Comparison of the present study and previous reports for detection of hydrazine

Probe Structure and reaction condition	Solvent System	LOD (ppb)	Linear Range (μM)	Possible interfering	Fluorescence strategy	Ref (Main Manuscript)
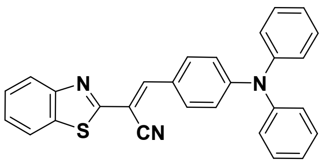 3 steps synthesis (EtOH, DMF)	DMSO-H_2O (10.0 μM HEPES buffer, 3: 7 v/v, pH = 7.4,)	6.6	0.75 – 1.5	None	ICT based Turn-on	Samanta et al. [2]
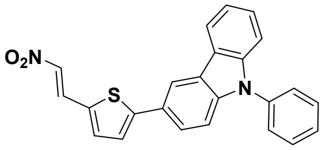 3 step synthesis (Nitromethane, DMSO)	DMSO/PBS solution (1/1; v/v, pH 7.4)	19.2	0 − 130	ClO^-	ICT based Turn-on	Wang et al. [3]
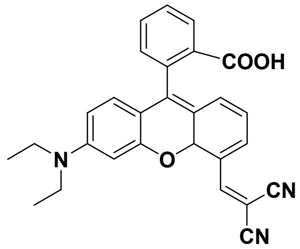 2 step synthesis (ACN, piperidine)	DMSO/PBS (v/v = 5:5, pH = 7.4)	25.6	0 – 10	CN^-	ICT based Turn-off	Mu et al. [4]
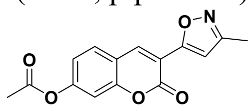 3 steps synthesis (EtOH, CH_2Cl_2)	CH_3CN/PBS (10 mM, pH = 7.10, 3/7, v/v)	2.90	0 – 50	None	AIE-ICT based Turn-on	Fan et al. [5]
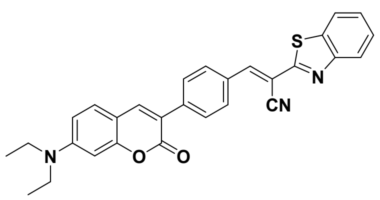 3 step synthesis (1,4-dioxane, EtOH)	1,4-dixoane/H_2O (10 mM, v/v = 9/1, pH = 7.4)	3.2	0 – 30	None	AIE-ICT based Turn-on	Lin et al. [6]

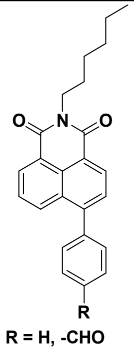 2 step synthesis (EtOH, THF:H_2O (3:1))	PBS Buffer (pH 7.4)	0.081	0 – 0.5	None	AIEE-ICT based Turn-off	Lyer et al. [7]
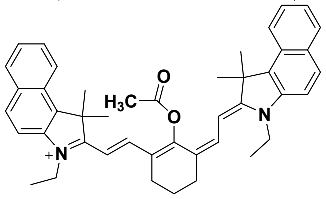 1 step synthesis (CH_2Cl_2)	HEPES (pH 7.4)	0.48	0 – 80	None	ICT based Turn-off	Yu et al. [8]
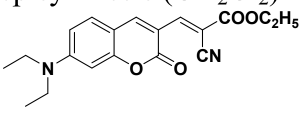 2 steps synthesis Solution phase (EtOH)	DMF:PBS Buffer (3:7, pH 7.4)	5.2	0 – 600	Bisulfite	Amination Based Turn-off	Su et al. [9]
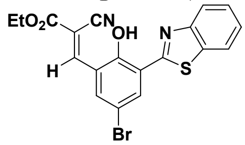 3 steps synthesis (H_2O_2, TFA, EtOH)	CH_3CN–H_2O (1:9, v/v)	13.8	0 – 25	None	ESIPT based Turn-on	Maiti et al. [10]
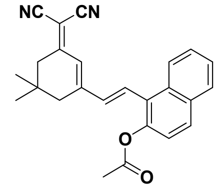 3 steps synthesis (EtOH, CH_2Cl_2)	DMSO	2.6	2 – 12	None	ESIPT based Turn-on	Yin et al. [11]
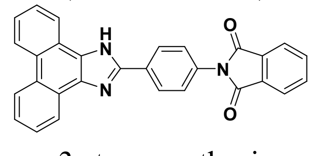 3 steps synthesis (THF-H_2O, CH_3COOH)	HEPES buffer (pH 7.2) using Triton X 100 surfactant	1.5	0 – 30	None	Cleavage by gabriel pathway (Turn-on)	[12]

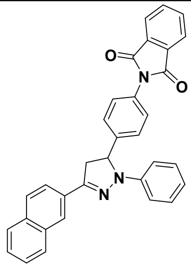 4 step synthesis (EtOH, ethyl acetate, acetic acid)	CH_3CN:PBS Buffer (30:70, pH 7.4)	2	10 – 20	None	Cleavage by Gabriel pathway (Turn-on)	Wan et al. [13]
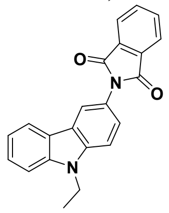 4 steps synthesis (DMF, AcOH, EtOH)	CH_3CN:HEPES Buffer (1:1, pH 7)	85.4	100 – 400	None	Cleavage by Gabriel pathway (Turn-on)	Hu et al. [14]
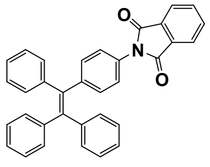 TPE-PMI 1 steps synthesis (mechanochemical, under neat condition)	CH_3CN:HEPES (3:97 v/v)	6.4	0.2 – 3	None	Cleavage by Gabriel pathway (Turn-on)	**Present Work**

12. Reference

1. A. Chatterjee, D. G. Khandare, P. Saini, A. Chattopadhya, M. S. Majik and M. Banerjee, *RSC Adv.,* 2015, **5**, 31479.
2. S. K. Samanta, K. Maiti, S. S. Ali, U. N. Guria, A. Ghosh, P. Datta and A. K. Mahapatra, *Dyes Pigm*., 2020, **173**, 07997.
3. L. Wang, Q. Pan, Y. Chen, Y. Ou, H. Li and B. Li, *Spectrochim. Acta Part A,* 2020, **241**, 118672.
4. S. Mu, H. Gao, C. Li, S. Li, Y. Wang, Y. Zhang, C. Ma, H. Zhang and X. Liu, *Talanta,* 2021, **221**, 121606.
5. H. Wu, Y. Wang, W.-N. Wu, Z.-Q. Xu, Z.-H. Xu, X.-L. Zhao and Y.-C. Fan, *Spectrochim. Acta Part A,* 2019, **222**, 117272.
6. X. Kong, M. Li, Y. Zhang, Y. Yin and W. Lin, *Sens. Actuators B,* 2021, **329**, 129232.
7. N. Meher, S. Panda, S. Kumar and P. K. Iyer, *Chem. Sci.,* 2018, **9**, 3978.

8. Y. Song, G. Chen, X. Han, J. You and F. Yu, *Sens. Actuators B,* 2019, **286**, 69.
9. J. Wu, J. Pan, J. Ze, L. Zeng and D. Su, *Sens. Actuators B,* 2018, **274**, 274.
10. S. Paul, R. Nandi, K. Ghoshal, M. Bhattacharyya and D. Maiti, *New J. Chem.,* 2019, **43**, 3303.
11. X. Shi, F. Huo, J. Chao, Y. Zhang and C. Yin, *New J. Chem.,* 2019, **43**, 10025.
12. F. H. A. Ali, N. Taye, D. G. Mogare, S. Chattopadhyay and A. Das, *Chem. Commun.,* 2016, **52**, 6166.
13. L. Wang, F.-Y. Liu, H.-Y. Liu, Y.-S. Dong, T.-Q. Liu, J.-F. Liu, Y.-W. Yao and X.-J. Wan, *Sens. Actuators B,* 2016, **229**, 441.
14. W.-D. Wang, Y. Hu, Q. Li and S.-L. Hu, *Inorg. Chim. Acta,* 2018, **477**, 206.

^{1}H NMR spectrum of TPE-PMI

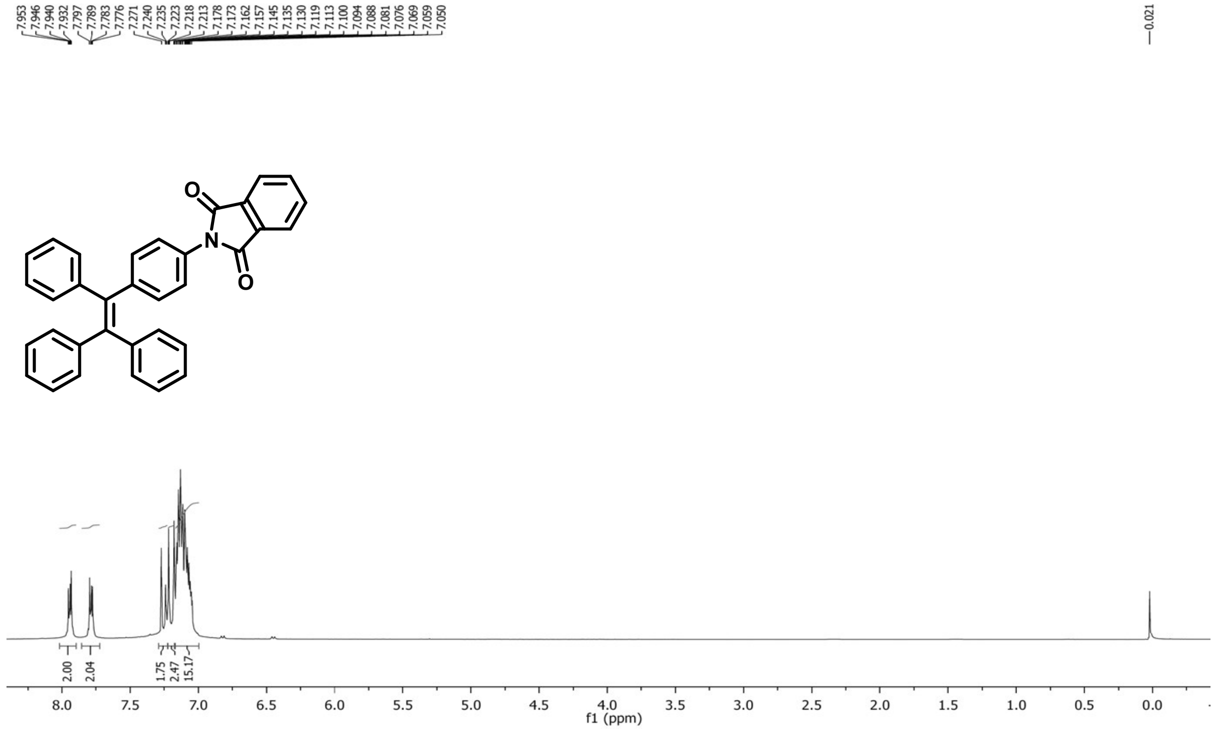

^{13}C NMR spectrum of TPE-PMI

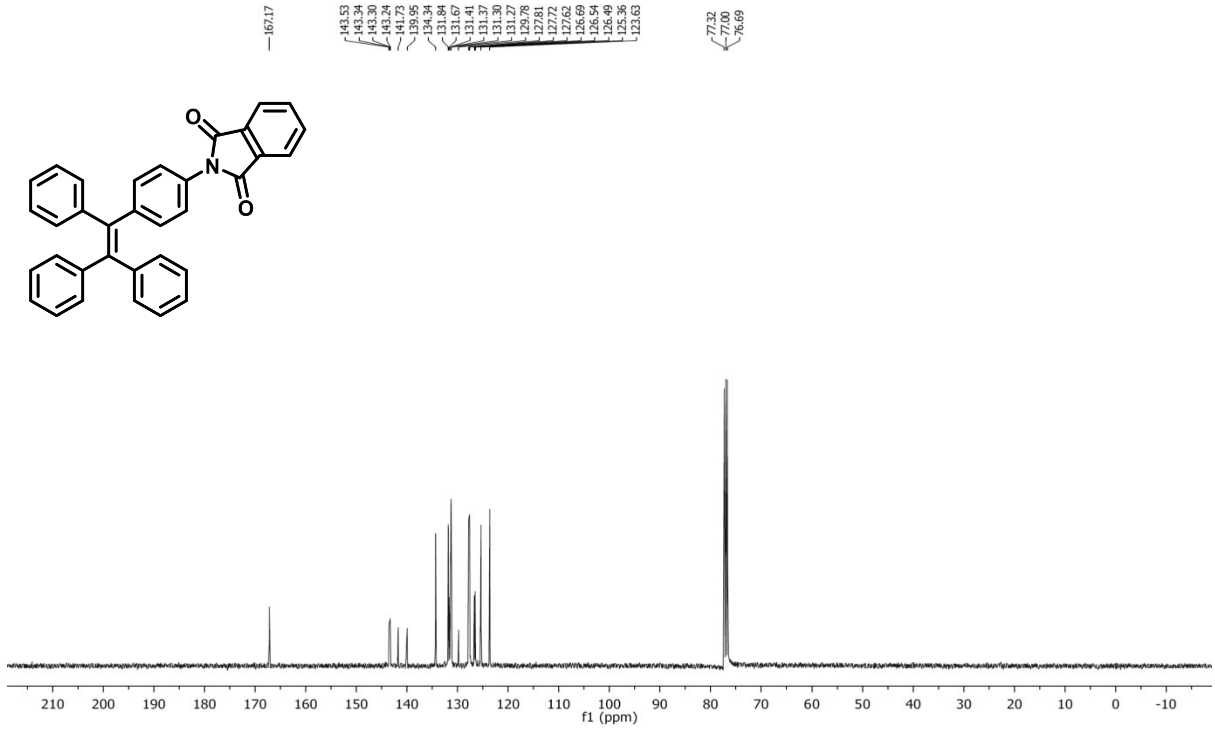

HRMS of TPE-PMI

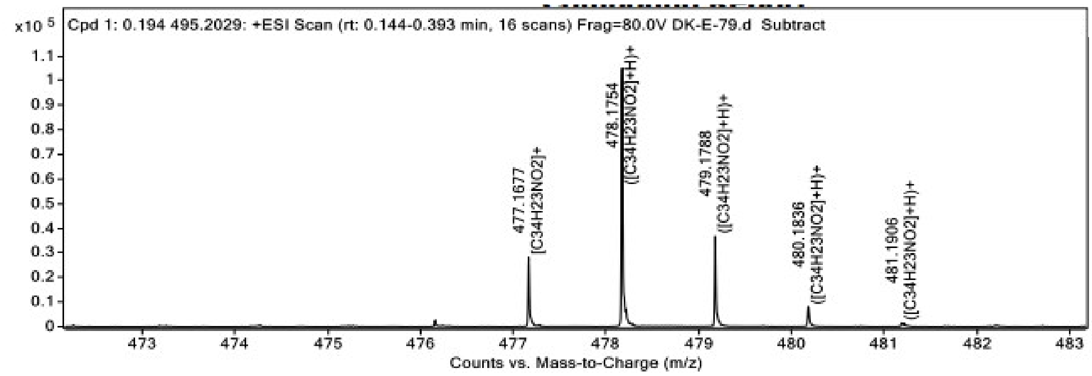

MS Spectrum Peak List

m/z	*Calc m/z*	Diff(ppm)	z	Abund	Formula	Ion
477.1677	477.1723	9.62	1	28294.27	C34H23NO2	M+
478.1754	478.1802	9.85	1	106468.26	C34H23NO2	(M+H)+
479.1788	479.1835	9.71	1	36869.72	C34H23NO2	(M+H)+
480.1836	480.1867	6.28	1	7980.82	C34H23NO2	(M+H)+
481.1906	481.1897	-1.91	1	1567.28	C34H23NO2	(M+H)+

IR spectrum of TPE-PMI

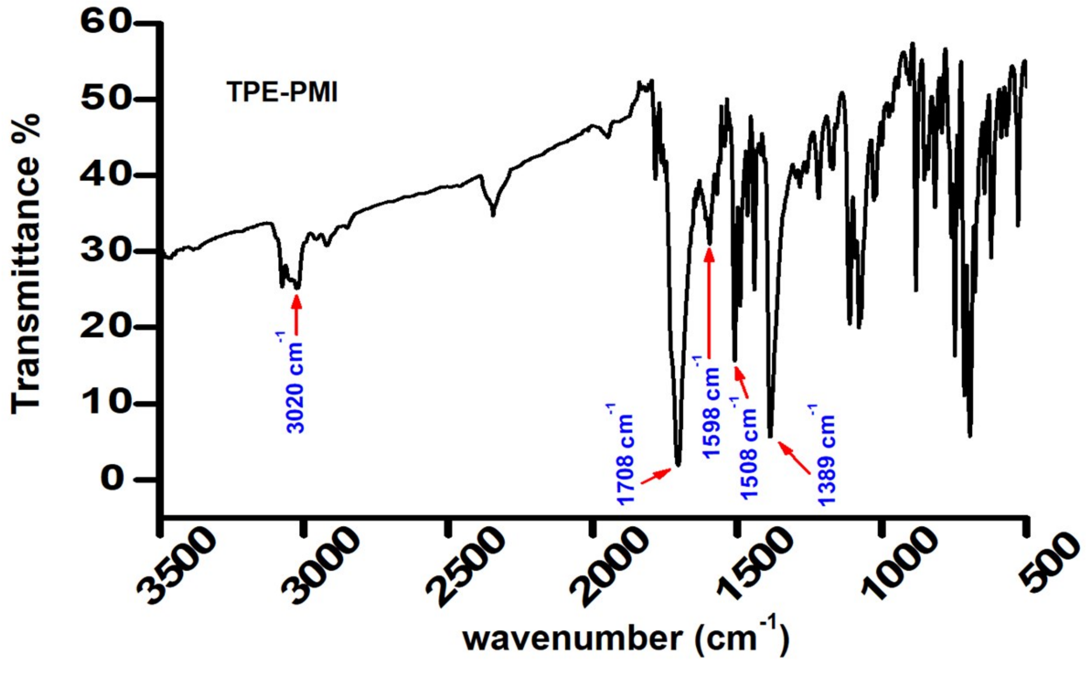

^{1}H NMR spectrum of TPE-NH_2

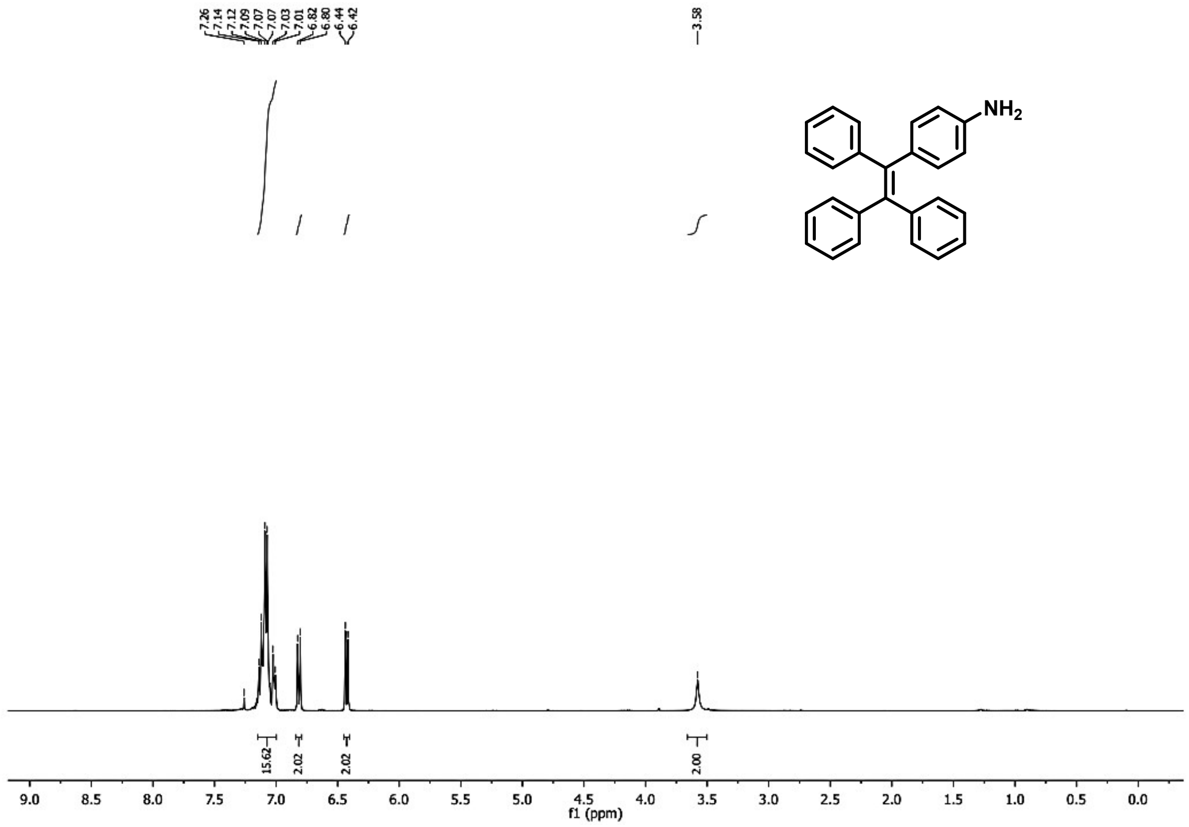

^{13}C NMR spectrum of TPE-NH_2

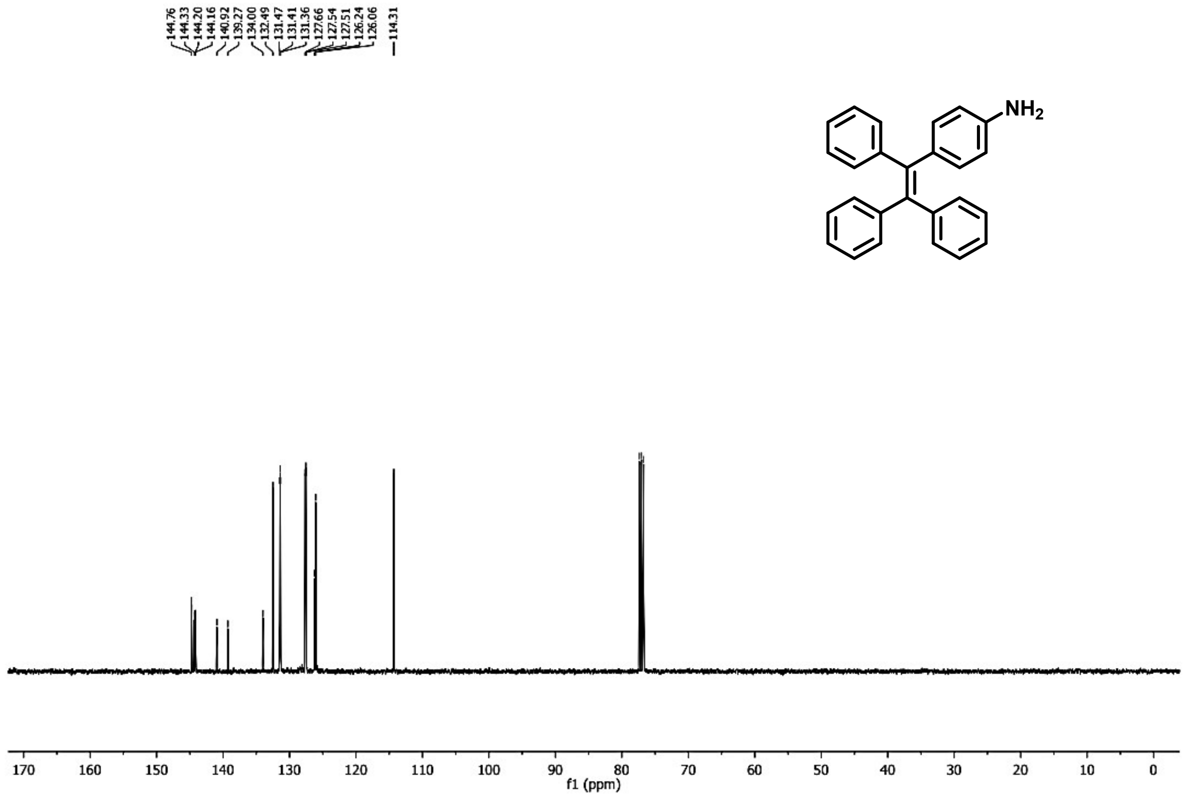
144.76
144.33
144.20
144.16
140.92
139.27
134.00
132.49
131.47
131.41
131.36
127.66
127.54
127.51
126.24
126.06
114.31
NH2
170
160
150
140
130
120
110
100
90
80
70
60
50
40
30
20
10
0
f1 (ppm)
